# Semaglutide-induced loss of skeletal muscle mass is blunted by co-administration of ketone esters

**DOI:** 10.1172/jci.insight.201810

**Published:** 2026-06-09

**Authors:** Yasser Abuetabh, Mya A. Schmidt, Masaaki Naganuma, Ramana Vaka, Mahmoud A. El-Ghiaty, Shelly Braun, Ethan A. Kwan, Matthieu C.P. Zolondek, Darius Sahid, Laibah Khan, Rajat K. Shandal, Ashley L. Trudeau, Yaning Li, Sufyan O. Malik, Qiuyu Sun, Danica K. Roth, Daniela Y. Morales-Llamas, Jody L. Levasseur, Mourad Ferdaoussi, Richard P. Fahlman, Jason R.B. Dyck

**Affiliations:** 1 Cardiovascular Research Centre,; 2 Alberta Diabetes Institute,; 3 Women and Children’s Health Research Institute,; 4 Department of Pediatrics,; 5 Department of Biochemistry, Faculty of Medicine & Dentistry, and; 6 Faculty Saint-Jean, University of Alberta, Edmonton, Alberta, Canada.

**Keywords:** Metabolism, Muscle biology, Obesity

## Abstract

While glucagon-like peptide-1 receptor agonists (GLP-1RAs) like semaglutide are effective in treating obesity, up to 45% of the resulting weight loss can be attributed to skeletal muscle loss. Given the critical role of skeletal muscle in health and mobility, this may have long-term adverse consequences. Herein we investigated whether oral ketone ester supplementation could prevent semaglutide-induced muscle loss and explored the underlying molecular mechanisms. Obese, glucose-intolerant mice received vehicle, semaglutide, or semaglutide plus a β-hydroxybutyrate–generating ketone ester for 3 weeks. Body composition, muscle strength, and endurance were assessed longitudinally. Semaglutide monotherapy reduced lean mass, impaired muscle strength, and suppressed mitochondrial gene expression while elevating atrophy-related genes in skeletal muscle samples. Co-administration with ketone ester preserved skeletal muscle mass and function without compromising fat loss. Mechanistically, ketone ester cotreatment prevented semaglutide-induced changes in mitochondrial and atrophy-related gene expression, suggesting that mitochondrial defects and impaired ketone metabolism contribute to GLP-1RA–induced muscle loss. Together, these findings demonstrate that ketone ester supplementation can maintain muscle mass and performance during semaglutide-driven weight loss. These preclinical findings support ketone therapy as a promising strategy to counteract the sarcopenia-promoting effects of GLP-1RAs and warrant clinical evaluation to assess its translational potential.

## Abstract 2

Ketone ester supplementation protects against muscle loss during semaglutide treatment without compromising fat reduction, offering a potential strategy to improve GLP-1 therapy outcomes.

## Introduction

Glucagon-like peptide-1 (GLP-1) receptor agonists (GLP-1RAs), such as semaglutide and liraglutide, have been approved for use in treating obesity. Although GLP-1RAs are having a significant beneficial impact on the health of millions of people worldwide, clinical trials report that GLP-1RAs result in the loss of lean mass that includes skeletal muscle (1). In fact, clinical data from the STEP1 trial indicate that as much as 45% of semaglutide-induced weight loss may come from lean mass (1). Although there is debate about whether the muscle loss linked to semaglutide falls within the typical range seen with diet-induced weight loss, preserving muscle remains important regardless of the method of weight reduction. Indeed, given the importance of skeletal muscle for overall health and mobility, it is possible that the loss of skeletal muscle may negatively impact semaglutide-treated patients in the longer term.

Although the extent and/or importance of semaglutide-induced muscle loss remain unresolved (2), researchers and pharmaceutical companies are intensifying efforts to uncover the mechanisms by which GLP-1RAs reduce skeletal muscle mass and to develop therapies that can prevent this effect (3–6). Given the multifaceted role that skeletal muscle plays in supporting physical well-being and quality of life (7), finding ways to prevent loss of skeletal muscle mass could greatly enhance the beneficial impact of the GLP-1RAs in the future. In the absence of pharmacological agents designed to prevent skeletal muscle loss, a joint advisory from the American College of Lifestyle Medicine, the American Society for Nutrition, the Obesity Medicine Association, and The Obesity Society has outlined several dietary approaches that should be adhered to in order to help prevent skeletal muscle loss in patients using semaglutide (8). However, these recommendations have shown limited success for preserving lean mass in practice, whether because of poor adherence, insufficient efficacy, or a combination of both. This highlights the need for new and sustainable strategies to preserve lean mass during semaglutide treatment.

While several pharmacological approaches to preserve skeletal muscle mass in patients on GLP-1RAs are under investigation (3–6), the long path to approval underscores the pressing need for timely treatment solutions. Accordingly, we have recently focused our efforts on studying the efficacy of ketone therapy in mitigating semaglutide-induced lean mass loss that we and others have previously reported (9–12). Ketone therapy involves the oral supplementation of a clinically tested ketone ester that results in elevated concentrations of circulating ketone bodies to levels that are within normal physiological ranges (13). Previous studies have shown that ketones play a major role in maintaining skeletal muscle mitochondrial function by promoting energy stability during reduced caloric intake and nutrient availability (14). Additionally, ketone therapy is efficacious in several other rodent models of muscle loss, including hind-limb unloading, stroke-related sarcopenia, aging, type 2 diabetes, cancer cachexia, and sepsis-induced muscle weakness (15–20). Moreover, we recently showed (11) that ketone ester co-therapy with semaglutide prevents semaglutide-induced loss of cardiac mass as well as reduced lean mass in obese mice, highlighting the impact that ketone therapy may have as a co-therapy to prevent semaglutide-induced loss of skeletal muscle mass.

Given our previous findings (11) as well as those from prior work showing that ketones play a major role in maintaining skeletal muscle mass in normal physiology (21) and during reduced caloric intake (14), the goal of this study was to investigate whether ketone therapy can preserve muscle mass during semaglutide treatment and to elucidate the molecular mechanisms underlying any observed benefit.

## Results

### In vivo assessment of ketone ester treatment in combination with semaglutide treatment of obese mice demonstrates prevention of semaglutide-induced loss of skeletal muscle mass.

**Figure 1:**
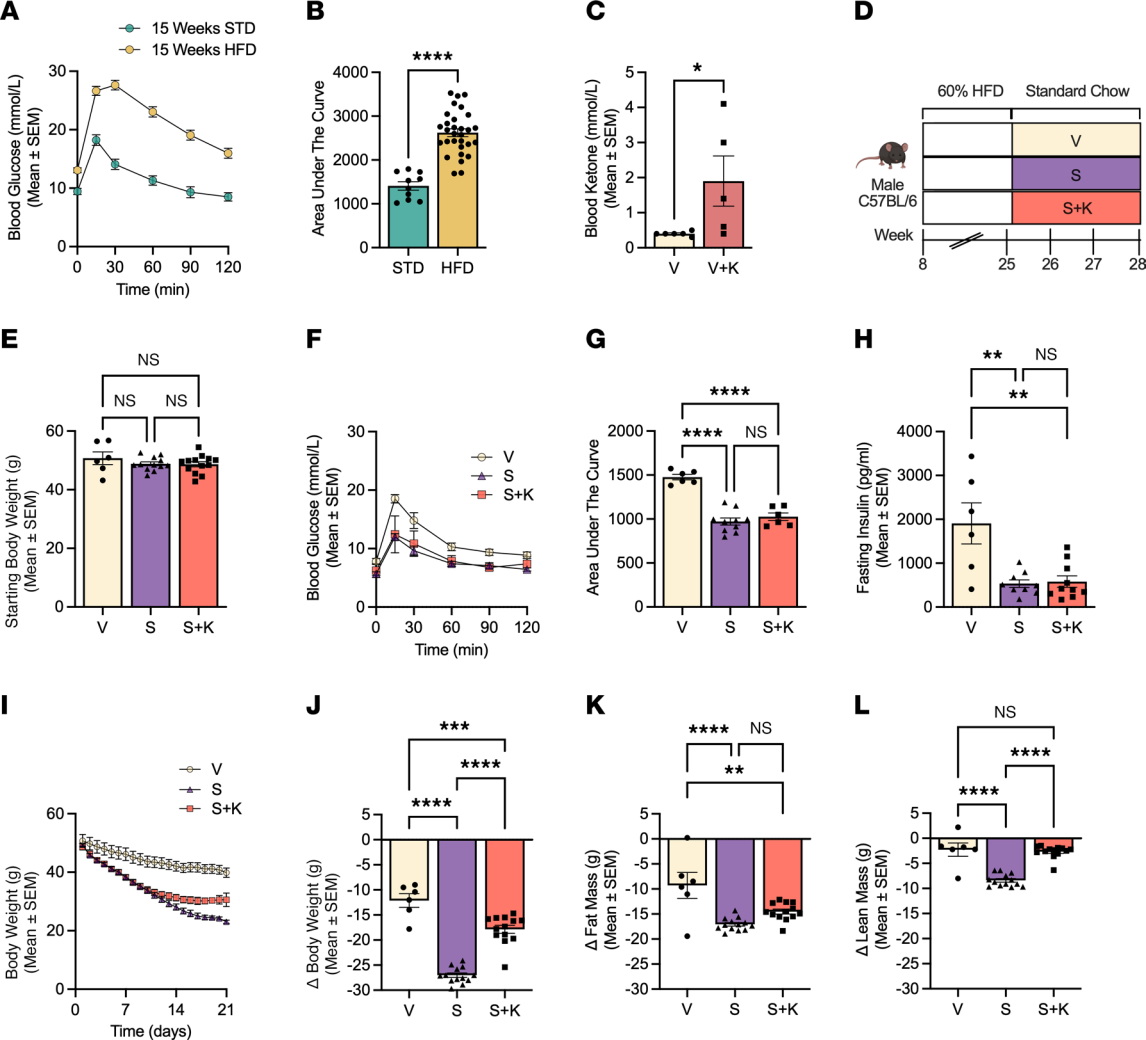
Ketone ester co-administration prevents semaglutide-induced lean mass loss without impacting fat loss. (**A**) Intraperitoneal glucose tolerance testing (IPGTT) was performed on male C57BL/6N and C57BL/6J mice after 15 weeks of high-fat diet (HFD) or standard chow (STD) (*n* = 10–30). (**B**) Glucose clearance represented by the area under the curve (AUC) of the IPGTT of **A**. (**C**) Blood β-hydroxybutyrate (ketone) concentrations were measured in obese mice following either vehicle administration (V) or twice-daily administration of 0.114 g/mL ketone ester in drinking water (V+K). (**D**) Schematic of the treatment timeline and group allocations. Created with BioRender.com. (**E**) The starting body weights of the studied groups, vehicle (V), semaglutide (S), and semaglutide combined with ketone ester (S+K). (**F**) IPGTT was repeated after 3 weeks of treatment with vehicle (V), semaglutide (S), or semaglutide combined with ketone ester (S+K) (*n* = 7–10). (**G**) Glucose clearance represented by the AUC of the IPGTT of **F**. (**H**) Fasting insulin levels. (**I**) Daily body weight measurements throughout the duration of the study. (**J**–**L**) Changes in body weight, fat mass, and lean mass were assessed following the 3-week intervention period using EchoMRI (*n* = 6–13). For comparisons among 3 groups, 1-way ANOVA was performed followed by Tukey’s multiple-comparison test (*P < 0.05; **P < 0.01; ***P < 0.001; ****P < 0.0001). All data are expressed as mean ± SEM. Data presented in this figure were obtained from experiments performed on male C57BL/6N and C57BL/6J mice.

Our previous work showing that lean mass is reduced by semaglutide treatment in obese mice was performed on obese mice fed a high-fat, high-sucrose diet consisting of 45% fat with no signs of glucose intolerance (22). Therefore, to investigate whether the same effects were observed in obese mice that were glucose intolerant, we fed 8-week-old male mice either a chow diet (control) or a diet consisting of 60% high fat for 15–17 weeks. To characterize the effects of this diet-induced obesity model, we show that over the 15–17 weeks of a high-fat diet, mice became obese (with an average body weight of 48.3 ± 0.6 g) and displayed significant glucose intolerance as assessed by an intraperitoneal glucose tolerance test (IPGTT; Figure 1, A and B). After confirmation of this model, our goal was to treat mice with semaglutide in the presence and absence of a ketone ester to determine whether ketone supplementation could prevent loss of skeletal muscle mass. Before treating the experimental cohort of mice, we first confirmed that the amount of ketone ester provided in the drinking water was sufficient to elevate β-hydroxybutyrate levels in the blood of obese mice to within physiologically normal levels (Figure 1C).

For the experimental cohort, after 15–17 weeks of a high-fat diet, the obese mice were switched to a regular chow diet to represent the 500-kcal-deficit-per-day changes of participants in the STEP1 trial (1) and randomly allocated to receive daily injections of either phosphate-buffered saline (PBS), semaglutide, or semaglutide plus ketone ester in their drinking water for 3 weeks (Figure 1D). Using this protocol, similarly obese mice from all 3 groups (Figure 1E) that were treated with semaglutide alone or in combination with the ketone ester displayed an expected improvement in IPGTT (Figure 1, F and G). Given that increased ketone levels in the blood are associated with progressive insulin resistance in humans (23) and that ketosis can initially increase insulin secretion that can lead to β cell exhaustion and hyperglycemia (24), we also confirmed that there were no differences in insulin concentrations in the semaglutide-treated versus the semaglutide + ketone ester–treated mice (Figure 1H). Additionally, mice were weighed daily, and we show that while vehicle-treated mice lost a small amount of body weight (BW), likely as a result of being switched to a regular chow diet, semaglutide-treated mice lost significantly more BW (Figure 1I). Interestingly, semaglutide + ketone ester–treated mice lost an amount of BW equivalent to the amount lost by the semaglutide-treated mice until day 12, at which point they lost no more BW for the remainder of the 21-day injection period. To investigate what contributed to this relative preservation of BW in semaglutide + ketone ester–treated mice at 21 days, we subjected mice to body composition analysis using EchoMRI to assess changes in lean and fat mass. Although semaglutide + ketone ester–treated mice lost less overall BW compared with those receiving semaglutide alone (Figure 1J), this difference was not attributable to alterations in fat mass reduction between treatment groups (Figure 1K), but rather to a marked preservation of lean mass in the semaglutide + ketone ester–treated group (Figure 1L). Together, these data show that the greater absolute BW loss in semaglutide- versus semaglutide + ketone ester–treated mice was driven by greater loss of lean mass, and that ketone administration effectively prevents semaglutide-induced lean mass loss.

### Ketone esters in combination with semaglutide treatment of obese mice result in improved skeletal muscle function.

**Figure 2:**
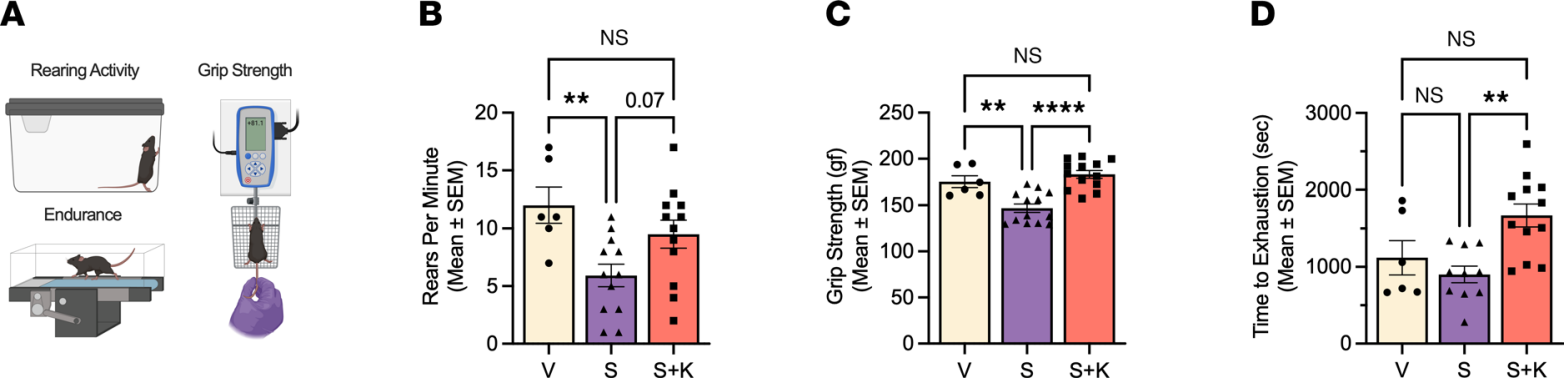
Ketone ester co-administration with semaglutide treatment prevents functional muscular decline and enhances physical performance. (**A**) Schematic depicting assessments of spontaneous rearing activity, grip strength, and treadmill endurance. Created with BioRender.com. (**B**) Rearing activity (*n* = 6–12). (**C**) Combined forelimb and hind-limb grip strength (*n* = 6–13). (**D**) Treadmill endurance (*n* = 6–12). Groups of mice are represented as vehicle (V), semaglutide (S), and semaglutide combined with ketone ester (S+K). Data presented in **B**–**D** were obtained from experiments performed on male C57BL/6N and C57BL/6J mice. For comparisons among 3 groups, 1-way ANOVA was performed followed by Tukey’s multiple-comparison test (**P < 0.01; ****P < 0.0001). All data are expressed as mean ± SEM.

In order to assess whether the preservation of lean mass resulting from ketone ester cotreatment with semaglutide results in improved skeletal muscle function in mice, we performed indirect skeletal muscle functional assays, including rearing activity, an all-limb grip strength test using a force gauge, and a running exercise performance test using a motorized treadmill (Figure 2A) as previously described (25). Firstly, our data demonstrate that mice treated with semaglutide had significantly reduced rearing activity compared with PBS-treated mice, and that this reduction showed a trend toward improvement in semaglutide-treated mice receiving ketone esters (Figure 2B). Additionally, combined forelimb and hind-limb grip strength tests revealed that grip strength was significantly reduced in semaglutide-treated mice compared with PBS control mice (Figure 2C) but that this reduction was prevented by co-administration of ketone esters (Figure 2C). Furthermore, time to exhaustion on the motorized treadmill did not differ between vehicle- and semaglutide-treated mice, likely because vehicle-treated mice remained obese while semaglutide-treated mice had reduced skeletal muscle mass (Figure 2D). Nevertheless, co-administration of semaglutide with ketone esters led to a significant improvement in the time to exhaustion in comparison with the other 2 groups (Figure 2D). Together, these data suggest that the preservation of skeletal muscle mass translates to improved muscle performance in semaglutide-treated mice that also received ketone ester supplementation.

### Direct assessment of skeletal muscle mass in semaglutide-treated obese mice in the absence and presence of ketone esters.

**Figure 3:**
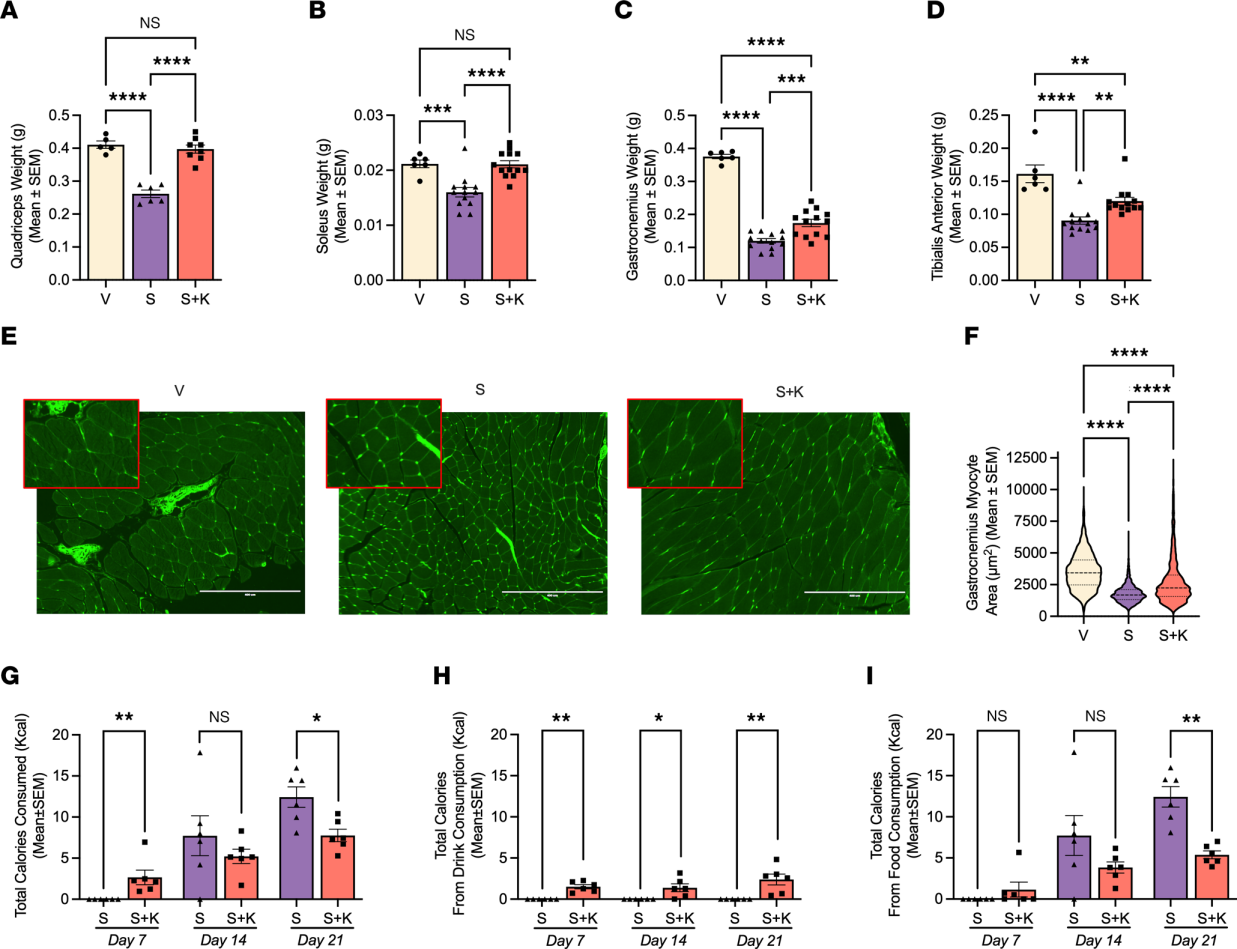
Reductions in skeletal muscle mass induced by semaglutide are blunted by ketone ester co-therapy. (**A**–**D**) Weights of the quadriceps, soleus, gastrocnemius, and tibialis anterior muscles were measured after 3 weeks of treatment (*n* = 6–13; data were obtained from experiments performed on male C57BL/6N and C57BL/6J mice). (**E**) Representative images of gastrocnemius muscle cross sections stained with wheat germ agglutinin. Scale bars: 400 μm; zoom, ×1.5. (**F**) Quantification of mean myocyte cross-sectional area in **E**. Data presented in **E** and **F** were obtained from experiments performed on male C57BL/6N mice. (**G**–**I**) Total calorie consumption (**G**), calories from drink consumption (**H**), and calories from food consumption (**I**) at 7, 14, and 21 days after initiation of the study protocol. Data presented in **G**–**I** were obtained from experiments performed on male C57BL/6J mice. Groups of mice are represented as vehicle (V), semaglutide (S), and semaglutide combined with ketone ester (S+K). Comparisons between 2 groups were conducted using an unpaired, nonparametric Mann-Whitney test. For comparisons among 3 groups, 1-way ANOVA was performed followed by Tukey’s multiple-comparison test (*P < 0.05; **P < 0.01; ***P < 0.001; ****P < 0.0001). All data are expressed as mean ± SEM.

To confirm that our in vivo assessment of lean mass was indeed reflective of the loss of skeletal muscle mass in our semaglutide-treated mice, we euthanized the mice and directly assessed the weight of individual skeletal muscle groups. Skeletal muscle from each mouse was isolated, and quadriceps, gastrocnemius, soleus, and tibialis anterior muscles were dissected and weighed. Given that tibia lengths were not different between groups (vehicle, 20.20 ± 0.21 mm; semaglutide, 19.97 ± 0.02 mm; semaglutide + ketone ester, 19.87 ± 0.03 mm), and thus there were no apparent effects on body length or structural proportions, muscle mass was reported in absolute values. As expected from a previous study (12), each of the 4 skeletal muscle groups in the semaglutide-treated mice demonstrated a significant reduction in mass in comparison with vehicle-treated mice. Strikingly, ketone ester cotreatment with semaglutide significantly preserved the mass of these muscles (Figure 3, A–D), with more dramatic effects observed in quadriceps and soleus muscles and less pronounced effects observed in the gastrocnemius and tibialis anterior muscles. Since there do not appear to be any specific commonalities between the 2 distinct pairs of muscle groups that have differential responses to ketone ester cotreatment, further studies are required to fully understand these differences. Nevertheless, these results parallel the preservation of lean mass and skeletal muscle function observed in our prior experiments (Figure 1L and Figure 2, B–D). In addition, histological analysis of the gastrocnemius muscle showed that semaglutide decreased myocyte surface area, while ketone ester cotreatment with semaglutide prevented these decreases even in the least responsive muscles (Figure 3, E and F). These data demonstrate that the reduced muscle mass in semaglutide-treated mice reflects smaller myocytes rather than decreases in intrafibrillar or intermuscular fat.

Lastly, to ascertain whether increased caloric intake in the semaglutide + ketone ester–treated mice could contribute to skeletal muscle sparing, we placed another cohort of obese mice in a Comprehensive Lab Animal Monitoring System at 7, 14, and 21 days after initiation of the study protocol to measure food and ketone ester consumption in order to ascertain differences in caloric intake between groups. Our data show that after 7 days, semaglutide-treated mice consumed very few calories (Figure 3G), whereas semaglutide + ketone ester–treated mice consumed modestly more calories, derived almost entirely from the ketone ester (Figure 3, H and I). Despite the differences in caloric intake between groups at this time point, lean muscle mass was not different between groups, suggesting that increased caloric intake in the semaglutide + ketone ester–treated mice was not contributing to skeletal muscle sparing in this group. In agreement with this, at days 14 and 21, when we observed the most muscle loss in semaglutide-treated mice, these mice had an overall higher caloric intake compared with semaglutide + ketone ester–treated mice (Figure 3I). Based on this, we conclude that the effects of the ketone ester in protecting skeletal muscle mass are likely not secondary to an elevated caloric intake.

### Semaglutide treatment of obese mice results in reduced BDH1 and SCOT expression in skeletal muscle.

**Figure 4:**
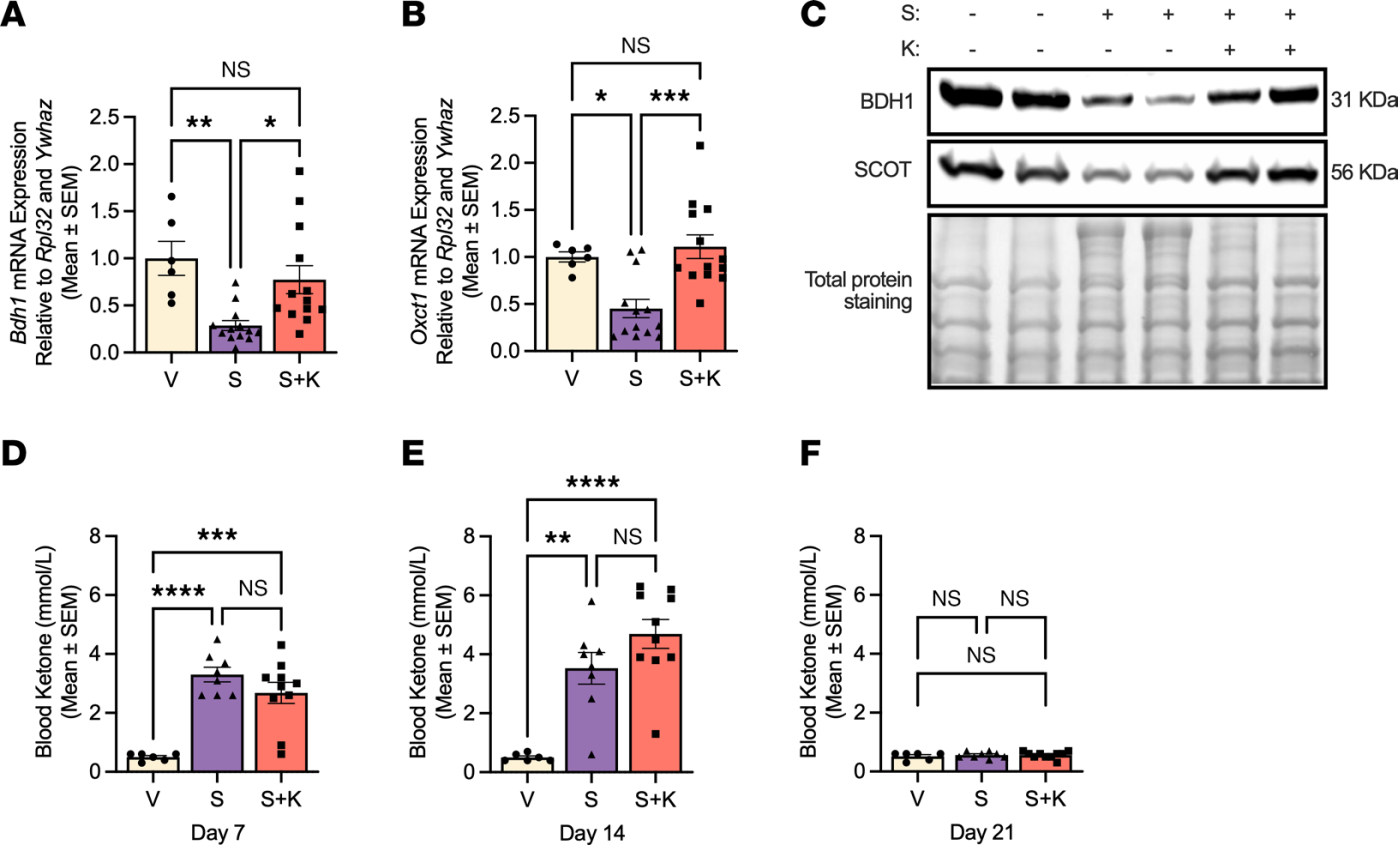
Semaglutide-induced loss of the ketolytic enzymes BDH1 and SCOT in gastrocnemius tissue is prevented by ketone ester co-therapy. (**A** and **B**) RT-qPCR data for *Bdh1* and *Oxct1* transcript expression in gastrocnemius tissue relative to *Rpl32* and *Ywhaz* (*n* = 6–13). (**C**) Representative immunoblot of gastrocnemius BDH1 and SCOT expression and total protein stain. Lysates from gastrocnemius muscle samples taken from groups of mice are represented as vehicle (V), semaglutide (S), or semaglutide combined with ketone ester (S+K). Total protein staining of the membrane is shown as a loading control. Data presented in **A**–**C** were obtained from experiments performed on male C57BL/6N and C57BL/6J mice. (**D**–**F**) Blood ketone levels were measured at 7 (**D**), 14 (**E**), and 21 (**F**) days after initiation of the study protocol. Data presented in **D**–**F** were obtained from experiments performed on male C57BL/6J mice. For comparisons among 3 groups, 1-way ANOVA was performed followed by Tukey’s multiple-comparison test (*P < 0.05; **P < 0.01; ***P < 0.001; ****P < 0.0001). All data are expressed as mean ± SEM. *Bdh1*, β-hydroxybutyrate dehydrogenase 1; *Oxct1*, 3-oxoacid CoA-transferase 1; *Rpl32*, ribosomal protein L32; *Ywhaz*, tyrosine 3-monooxygenase/tryptophan 5-monooxygenase activation protein ζ.

Given our recent findings that semaglutide treatment results in a downregulation of β-hydroxybutyrate dehydrogenase 1 (BDH1) expression in cardiac muscle and that this contributes to the loss of cardiac muscle mass (11), we speculated that this also occurs in skeletal muscle. To investigate this, we assessed BDH1 expression in gastrocnemius muscle from mice from all 3 treatment groups euthanized at 3 weeks after treatment. Consistent with our previous findings (11), we show that *Bdh1* transcript levels and BDH1 protein expression were significantly downregulated in skeletal muscle from mice treated with semaglutide (Figure 4, A and C). Additionally, we found that the rate-limiting ketolytic enzyme succinyl-CoA:3-ketoacid-CoA transferase (SCOT; gene nomenclature: *Oxct1*) was significantly downregulated at both the protein and mRNA levels in semaglutide-treated mice (Figure 4, B and C). Importantly, semaglutide-induced downregulation of both enzymes was blunted by co-administration of ketone esters, and protein abundance remained at baseline levels (Figure 4, A–C). Building on previous findings that BDH1 expression is required to maintain skeletal muscle mitochondrial function and support energy stability during caloric restriction and nutrient scarcity (14), along with recent reports linking downregulation of *Oxct1* and *Bdh1* to mitochondrial dysfunction in sarcopenia (26, 27), we propose that reduced BDH1 and SCOT expression may contribute to semaglutide-induced skeletal muscle loss and thus represent a key mechanism through which ketone ester supplementation preserves muscle mass.

Given our previous findings in mice with skeletal muscle–specific SCOT1 knockout showing that decreasing of ketolytic enzymes in skeletal muscle is a major driver of elevations in blood ketone levels (28), we also speculated that decreased skeletal muscle ketolytic enzymes could influence circulating blood β-hydroxybutyrate levels. In addition, since the semaglutide-induced dramatic and rapid loss of adipose tissue elevates fatty acids in the blood that act as substrates for the liver to synthesize ketones, we also hypothesized that these skeletal muscle and adipose tissue effects would significantly alter overall blood ketone levels, regardless of ketone ester supplementation. To address this, we measured circulating blood β-hydroxybutyrate levels in all 3 groups of mice at 7, 14, and 21 days after initiation of the study protocol. As expected, likely driven by a combination of the increased adipocyte lipolysis and reduction of skeletal muscle BDH1 and SCOT in semaglutide-treated mice, we observed a high level of ketones at 7 and 14 days after initiation of the weight loss period (Figure 4, D and E). Interestingly, despite ketone ester supplementation in the semaglutide + ketone ester–treated group of mice, we only observed a further elevation in blood β-hydroxybutyrate levels at 14 days of treatment (Figure 4, D and E). Strikingly, at 21 days after treatment, the concentration of β-hydroxybutyrate in the blood returned to baseline levels in both semaglutide- and semaglutide + ketone ester–treated groups of mice (Figure 4F). While we do not provide unequivocal data as to why this occurs, we speculate that the majority of the ketones at days 7 and 14 originated from elevated adipose-derived fatty acids that were used for hepatic ketogenesis and then secreted into the circulation. However, by day 21, adipocyte lipolysis is greatly reduced as BW stabilizes at low levels and there is a reduction in fatty acids available for hepatic ketogenesis. To what extent the reductions in skeletal muscle BDH1 and SCOT levels contribute to these changes in ketone levels in the blood has yet to be fully elucidated.

### Transcriptomic analysis of skeletal muscle in semaglutide-treated obese mice in the absence and presence of ketone esters.

**Figure 5:**
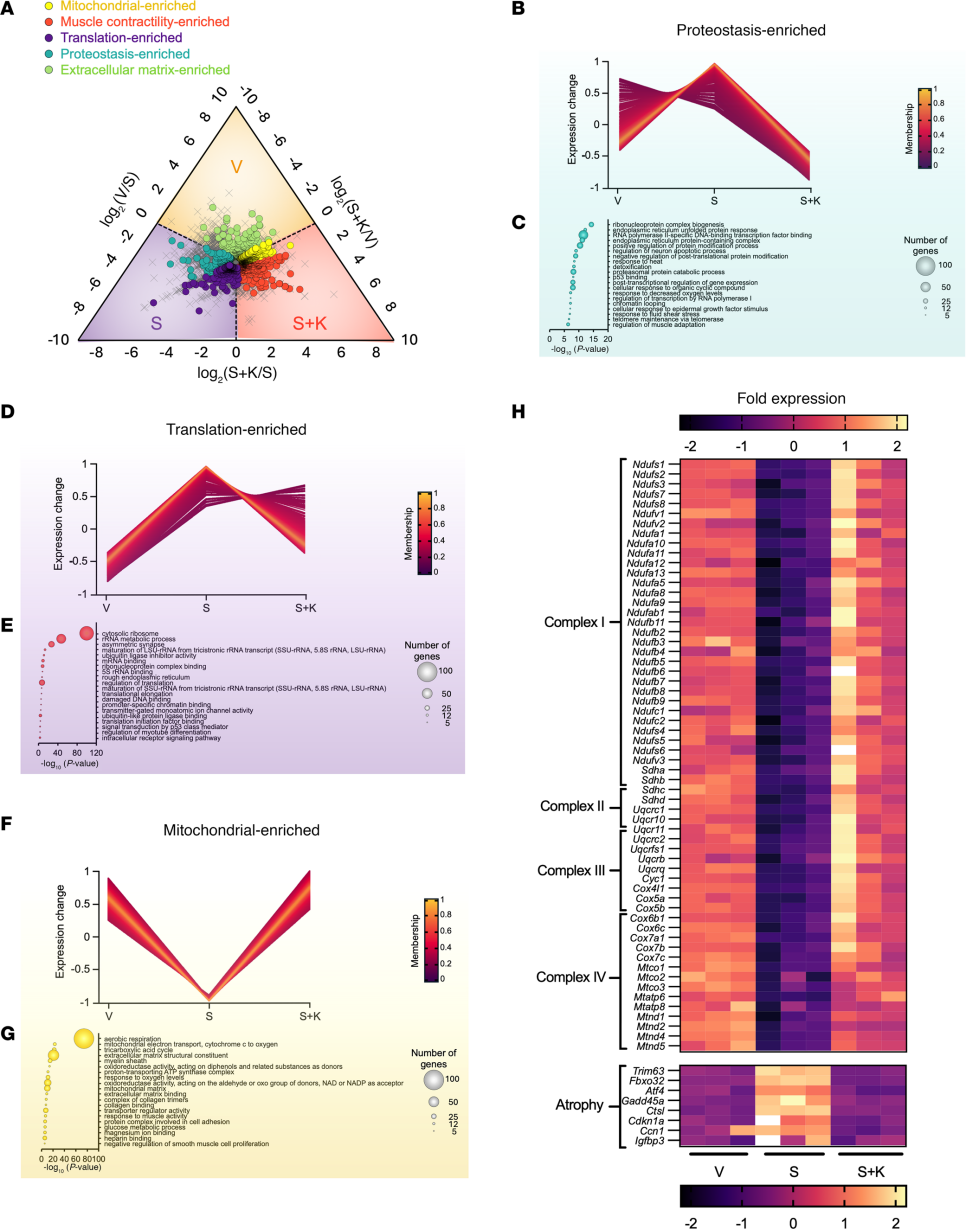
Ketone ester co-therapy prevents semaglutide-induced transcriptomic shifts in skeletal muscle involving proteostasis, translation, and mitochondrial pathways. (**A**) A triplot depicting log_2_ fold changes in gene expression across vehicle (V), semaglutide (S), and semaglutide + ketone ester (S+K) treatment groups. Genes highlighted in color are significantly altered (*P* < 0.05, fold change > 1.5), while gray crosses represent genes that are not significantly altered as assessed by ANOVA analysis. Genes are grouped into 5 distinct clusters based on expression trajectories: mitochondrial-enriched (yellow), muscle contractility–enriched (orange), translation-enriched (purple), proteostasis-enriched (teal), and extracellular matrix–enriched (green). The membership scale denotes how close to the trend each gene falls within the cluster. (**B**–**G**) Soft clustering of differentially expressed genes showing relative gene expression trends of interest: proteostasis-enriched cluster (**B**), translation-enriched cluster (**D**), and mitochondrial-enriched cluster (**F**), across V, S, and S+K conditions. Gene Ontology (GO) analysis presented in dot plots is located below differentially expressed gene cluster graphs (**C**, **E**, and **G**). Bubble size reflects the number of genes associated with each GO term; *x* axis indicates statistical significance as –log_10_ (*P* value). (**H**) Heatmaps of mitochondrial electron transport chain– and atrophy-related genes (*n* = 3). RNA-seq data were processed via 1-way ANOVA (*P* < 0.05, fold change ≥ 1.5). Heatmaps were generated using row-wise *z* score normalization of expression values. Data presented in the figure were obtained from experiments performed on male C57BL/6N mice.

To more fully characterize the physiological, biological, and cellular changes that were occurring in the skeletal muscle of obese mice treated with semaglutide or semaglutide + ketone ester, we performed transcriptomic analysis using RNA-seq data of gastrocnemius muscle from the 3 relevant groups of mice (vehicle, semaglutide, and semaglutide + ketone ester) at the end of the 3-week treatment period. Firstly, to examine how transcript abundance varied across the 3 treatment groups, we visualized gene-level expression using a triplot (Figure 5A). Statistically significantly altered genes (1-way ANOVA, *P* < 0.05, fold change > 1.5) are represented in the triplot, where each gene’s position reflects its relative expression across conditions. Genes at the center of the plot exhibit identical expression across all 3 groups, and genes closer to the apex of the triangle are highly expressed in one group compared with the others. Soft clustering identified multiple expression patterns, with 5 more dominant changes observed in clusters consisting of transcripts that were (a) mitochondrial-enriched, (b) muscle contractility–enriched, (c) translation-enriched, (d) proteostasis-enriched, and (e) extracellular matrix–enriched (Figure 5A). Overall, these changes represent important pathways that have previously been shown to be regulated in semaglutide-induced loss of skeletal muscle mass in mice (12).

### Cluster analysis of skeletal muscle transcripts in semaglutide-treated obese mice in the absence and presence of ketone esters.

Three clusters were selected for representation based on their expression profiles, relevance to a previous study (12), and relevance to the treatment response (Figure 5, B, D, and F). These clusters included transcripts that were either suppressed or elevated by semaglutide treatment, with expression patterns returning to vehicle-like levels in the semaglutide + ketone ester group. The gene cluster showing downregulated genes in the semaglutide + ketone ester combination treatment group compared with semaglutide treatment alone contained a proteostasis-enriched set of genes. Particularly, the Gene Ontology term “proteasomal protein catabolic process,” containing 29 genes, suggests that the combination treatment may help with lowering expression of genes related to muscle atrophy and wasting when ketone esters are present (Figure 5, B and C). Genes in the translation-enriched cluster that were elevated in semaglutide-treated mice were enriched for mainly translation-related terms among others (Figure 5, D and E), suggesting that the semaglutide treatment may potentially be causing an increase in transcripts involved in transcription and translational machinery in an apparent futile attempt to compensate for the atrophied muscle. Strikingly, 27% of the total significantly altered genes that were repressed in the semaglutide treatment group (Figure 5F) and normalized in the semaglutide + ketone ester group were categorized in the mitochondrial-enriched cluster (Figure 5G). This trend suggests that ketone ester cotreatment may partially prevent the transcriptomic phenotype induced by semaglutide, maintaining gene expression of many mitochondrial genes at baseline levels.

To extrapolate on the findings of transcript-level trends representing mitochondrial- and proteostasis-related processes, visualization of the expression patterns of genes related to mitochondrial electron transport chain (ETC) and to canonical atrophy was further presented in a heatmap format (Figure 5H). Semaglutide treatment led to broad suppression of ETC gene expression across complexes I–IV, supporting a transcriptional downregulation of mitochondrial bioenergetics. Importantly, ketone ester cotreatment largely preserved expression across these complexes (Figure 5H). Similarly, atrophy-associated transcripts that were elevated by semaglutide were markedly reduced with ketone ester cotreatment, reinforcing the protective effect of ketones on muscle integrity and mitochondrial function (Figure 5H). Together, these data show an expression pattern consistent with dramatic changes induced by semaglutide that are prevented by the addition of the ketone ester treatment in combination with semaglutide.

### The putative role of the mitochondria in contributing to the loss of skeletal muscle in semaglutide-treated obese mice.

**Figure 6:**
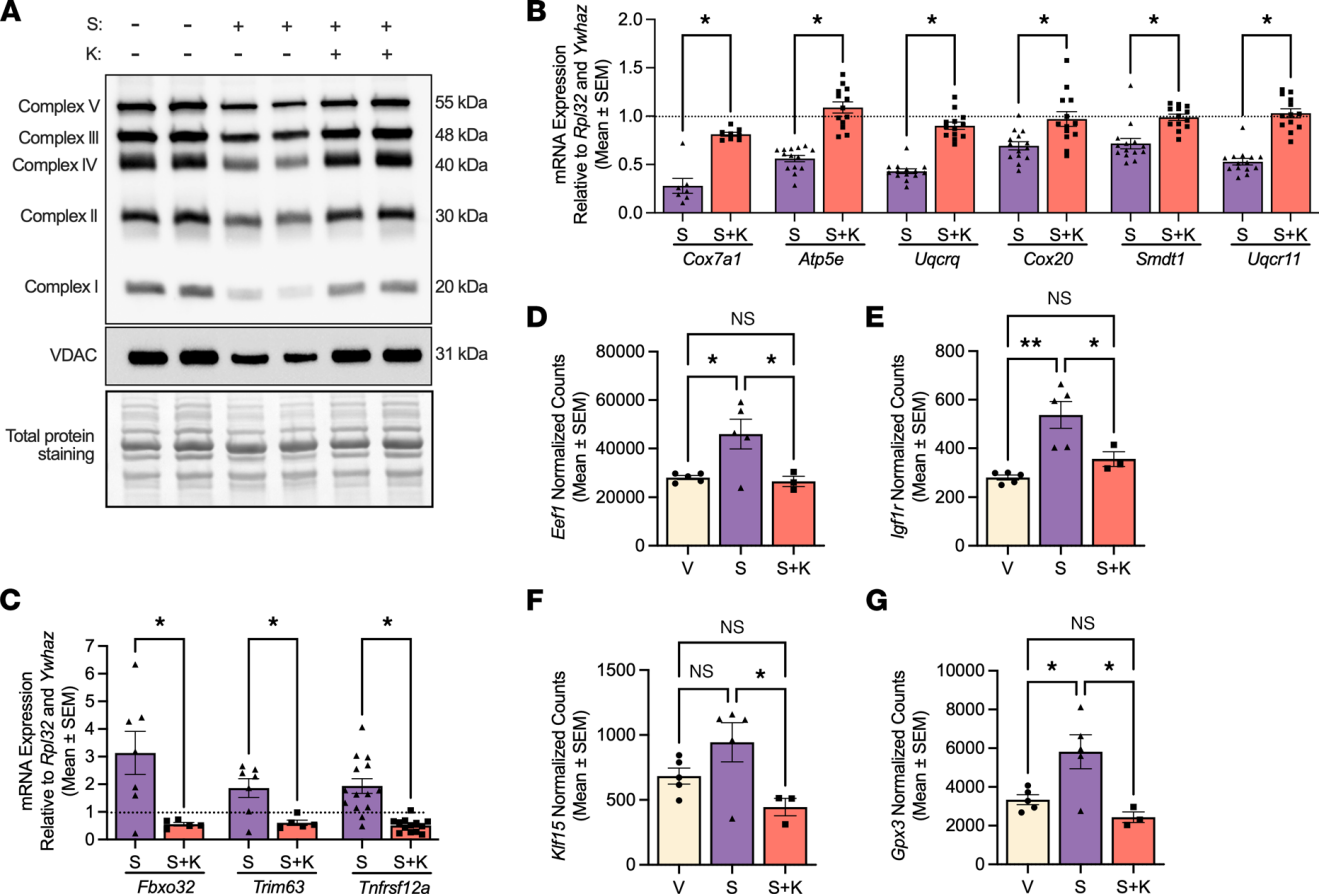
Ketone ester supplementation prevents mitochondrial OXPHOS complex protein expression and gene expression while suppressing atrophy- and stress-associated transcripts in semaglutide-treated muscle. (**A**) Representative immunoblot analysis of the mitochondrial OXPHOS complexes (I–V) and anti–voltage-dependent anion channel (anti-VDAC) from lysates of gastrocnemius muscle samples taken from groups of mice (C57BL/6N and C57BL/6J) represented as vehicle (V), semaglutide (S), or semaglutide combined with ketone ester (S+K). Total protein staining of the membrane is shown below as a loading control. (**B**) Gastrocnemius transcript expression of the mitochondrial electron transport–related genes *Cox7a1*, *Atp5e*, *Uqcrq*, *Cox20*, *Smdt1*, and *Uqcr11* normalized to *Rpl32* and *Ywhaz*, relative to vehicle-treated mice (*n* = 10–14). Dotted line indicates data for vehicle controls. (**C**) Gastrocnemius transcript expression of the atrophy-related genes *Fbxo32*, *Trim63*, and *Tnfrsf12a* normalized to *Rpl32* and *Ywhaz*, relative to vehicle-treated mice (*n* = 5–14). Dotted line indicates data for vehicle controls. Data presented in **B** and **C** were obtained from experiments performed on male C57BL/6N and C57BL/6J mice. (**D**–**G**) RNA-seq–derived normalized counts for *Eef1*, *Igf1r*, *Klf15*, and *Gpx3* (*n* = 3–5). Data presented in **D**–**G** were obtained from experiments performed on male C57BL/6N mice. Comparisons between 2 groups were conducted using an unpaired, nonparametric Mann-Whitney test. For comparisons among 3 groups, 1-way ANOVA was performed followed by Tukey’s multiple-comparison test (*P < 0.05; **P < 0.01). Total normalized counts were compared using 1-way ANOVA followed by Tukey’s multiple-comparison post hoc test. A value of *P* < 0.05 was considered statistically significant. All data are expressed as mean ± SEM. *Cox7a1*, cytochrome *c* oxidase subunit 7A1; *Atp5e*, ATP5F1E ATP synthase F1 subunit ε; *Uqcrq*, ubiquinol–cytochrome *c* reductase, complex III subunit VII; *Cox20*, cytochrome *c* oxidase assembly factor; *Smdt1*, single-pass membrane protein with aspartate-rich tail 1; *Uqcr11*, ubiquinol–cytochrome *c* reductase, complex III subunit XI; *Fbxo32*, atrogin-1; *Trim63*, muscle RING-finger protein 1; *Tnfrsf12a*, TNF-related weak inducer of apoptosis; *Rpl32*, ribosomal protein L32; *Ywhaz*, tyrosine 3-monooxygenase/tryptophan 5-monooxygenase activation protein ζ; *Eef1*, eukaryotic elongation factor 1 gene; *Igf1r*, insulin-like growth factor 1 receptor gene; *Klf15*, Krüppel-like factor 15 gene; *Gpx3*, glutathione peroxidase 3 gene.

Our transcriptomics analysis in skeletal muscle identified significant changes in transcript levels of genes that encode for proteins involved in the mitochondrial ETC between the 3 treatment groups, suggesting that mitochondrial function and/or number may play an important role in governing the loss of skeletal muscle mass. Indeed, dysfunctional mitochondria are widely accepted as contributing to the etiology of sarcopenia (29), and it has been suggested that this impaired mitochondrial function and the resulting reduction in ATP production limit the cellular processes that are necessary to maintain muscle mass (29). Consistent with these previous findings and our transcriptomic analysis of ETC-related genes, we further show that the protein expression of all 5 of the mitochondrial oxidative phosphorylation (OXPHOS) complexes (I–V) was decreased in the gastrocnemius muscles of semaglutide-treated mice compared with vehicle controls (Figure 6A). In addition, we show that all of the OXPHOS complexes were unaltered in the gastrocnemius muscles of the semaglutide + ketone ester treatment group and were expressed at similar levels to those in the vehicle-treated mice (Figure 6A). Moreover, we also show that the outer mitochondrial membrane voltage-dependent anion channel (VDAC) was decreased in the gastrocnemius muscles of semaglutide-treated mice but was maintained at normal levels in gastrocnemius muscles of semaglutide + ketone ester–treated mice (Figure 6A). Based on the fact that components of complexes I, III, IV, and V are encoded by the mitochondria and VDAC levels can be used as a marker of mitochondrial number (30), these findings suggest reduced mitochondrial number in skeletal muscle of semaglutide-treated mice, which is preserved in the semaglutide + ketone ester–treated group of mice.

Although we provide evidence that a reduction in mitochondrial number may account for some of the reduction in skeletal muscle mass in semaglutide-treated mice, it is possible that impaired mitochondrial function also plays a role. Consistent with this, after a literature search to identify mitochondrial proteins that have been shown to be involved in maintaining skeletal muscle mass, we also measured the transcript levels of the gene encoding cytochrome *c* oxidase subunit 7A1 (*Cox7a1*), as it was one of the most dramatically decreased transcript levels resulting from semaglutide treatment (Figure 6B) compared with vehicle (dotted black line). COX7A1 is a mitochondrial protein that is necessary for mitochondrial complex IV dimer stabilization and appears to be essential for mediating mitochondrial OXPHOS activity necessary for skeletal muscle maturation, with the loss of COX7A1 resulting in a reduction in skeletal muscle mass and performance (31). Importantly, ketone ester cotreatment of semaglutide-treated obese mice can partially normalize *Cox7a1* expression (Figure 6B) and thus may contribute to preserved skeletal muscle mass induced by ketone therapy.

In agreement with OXPHOS activity being reduced in skeletal muscle from obese mice treated with semaglutide, we also show that a cluster of 5 genes that encode mitochondrial proteins that contribute to OXPHOS and ATP production (ATP5F1E ATP synthase F1 subunit ε [*Atp5e*]; ubiquinol–cytochrome *c* reductase, complex III subunit VII [*Uqcrq*]; cytochrome *c* oxidase assembly factor [*Cox20*]; single-pass membrane protein with aspartate-rich tail 1 [*Smdt1*]; and ubiquinol–cytochrome *c* reductase, complex III subunit XI [*Uqcr11*]) were reduced in skeletal muscle of semaglutide-treated mice (Figure 6B). This cluster of genes is particularly interesting as they have been shown to be specifically regulated by β-hydroxybutyrylation, an epigenetic modification essential in the reversal of age-associated sarcopenia (17). Importantly, we also show that ketone therapy during semaglutide treatment prevented the decrease in these transcript levels (Figure 6B). Taken together, these data provide compelling evidence that changes in transcript levels of multiple genes that encode mitochondrial proteins that contribute to OXPHOS and ATP production in skeletal muscle may contribute to semaglutide-induced loss of skeletal muscle mass.

### The potential involvement of atrophy in the regulation of muscle mass in semaglutide-treated obese mice.

As there is strong evidence showing that the mitochondrial dysfunction and increased atrophy in skeletal muscle are tightly coupled (32, 33), we next wanted to investigate whether this was also the case in our semaglutide-treated mice. We observed a significant increase in transcript levels of key atrophic regulatory proteins that govern skeletal muscle atrophy via the ubiquitin-proteasome system, such as atrogin-1 (encoded by the *Fbxo32* gene), muscle RING-finger protein 1 (MuRF-1; encoded by the *Trim63* gene), and TNF-related weak inducer of apoptosis (TWEAKR; encoded by the *Tnfrsf12a* gene), in gastrocnemius muscle of semaglutide-treated mice (Figure 6C). Since these proteins are central components of signaling pathways that promote muscle wasting and contribute to skeletal muscle pathology when upregulated (34–36), their increased expression is consistent with the reduced skeletal muscle mass observed in semaglutide-treated mice. Moreover, many of these same genes were also recently shown by another group to be increased in skeletal muscle from mice treated with semaglutide (12), further supporting our findings. More importantly, when mice were treated with semaglutide + ketone esters, the expression of these genes remained at or below control levels (Figure 6C). While these transcriptional data alone are insufficient to definitively conclude that semaglutide activates molecular pathways driving skeletal muscle atrophy or that ketone therapy directly prevents this, these findings align with the physiological changes observed in skeletal muscle mass in these mice.

### The potential involvement of additional ketone signaling pathways in the regulation of muscle mass in semaglutide-treated obese mice.

A recent study found that semaglutide upregulates transcriptional expression of the eukaryotic elongation factor 1 gene (*Eef1*) and the insulin-like growth factor 1 receptor gene (*Igf1r*), which encode proteins associated with protein translation and muscle size (12). Consistent with this, our analysis of normalized RNA-seq counts revealed increased expression of both of these genes in semaglutide-treated mice, which was attenuated by co-administration of ketone esters (Figure 6, D and E). The aforementioned study also found that the Krüppel-like factor 15 gene (*Klf15*), which encodes a key regulator of muscle protein balance that is upregulated during muscle atrophy, is increased with semaglutide treatment (12). In agreement with this, we observed a trend toward elevated *Klf15* transcript levels in semaglutide-treated mice, an effect that was abrogated with ketone ester cotreatment (Figure 6F). Additionally, the glutathione peroxidase 3 (*Gpx3*) gene, which encodes a protein involved in the glutathione antioxidant pathway, was previously shown to be upregulated by semaglutide (12). We similarly found a significant increase in *Gpx3* expression in semaglutide-treated mice, which was reversed with ketone ester co-administration (Figure 6G). Together, these findings suggest that the semaglutide-induced changes in protein balance as well as oxidative stress are completely normalized in the presence of ketones esters.

## Discussion

Despite the findings in clinical trials that semaglutide-induced loss of lean mass contributed up to 45% of patients’ total weight loss, it is not clear how much of this can be attributed to skeletal muscle loss or whether this has a direct impact on physical activity. Studying this latter effect in humans is challenging, as significant fat loss often improves physical performance in the short term, potentially masking any immediate deleterious effects of skeletal muscle loss that may become more pronounced over time. However, in our mouse model of semaglutide-induced weight loss, we were able to assess muscle performance and capture some of these negative effects, as well as directly measure the extent of the loss of skeletal muscle mass via gravimetric analysis of excised muscle groups. In this study, we confirm that a significant amount of skeletal muscle is lost in response to semaglutide treatment (12) and provide additional data showing that this adversely impacts skeletal muscle performance in functional assays of spontaneous activity, strength, and exercise tolerance. More importantly, we show that supplementing mice with a ketone ester during semaglutide treatment effectively prevents semaglutide-induced loss of skeletal muscle mass without impacting magnitude of adipose loss. If these beneficial effects translate to humans treated with semaglutide, ketone ester co-administration could represent a safe and practical strategy to enhance the safety of GLP-1RAs by preserving muscle mass without compromising adipose tissue loss. The long-term consequences of this could be substantial, as low muscle mass is an independent predictor of poor health outcomes associated with physical limitations, including poor quality of life and shorter survival (7), making muscle mass preservation during treatment clinically meaningful.

Although the current study has identified potential mechanisms that may contribute to the semaglutide-induced loss of skeletal muscle mass, as well as explain how ketone therapy prevents this, the precise mechanism(s) of action of this prevention has yet to be fully elucidated. Indeed, the mechanism may consist of simply providing an additional energy source that results in glucose sparing and subsequent preservation of muscle-derived amino acids needed for energy production. Since we show that the total calories consumed (via food and/or via ketone drink) were lower in the semaglutide plus ketone ester group compared with the semaglutide monotherapy group, these calorie-mediated muscle-sparing effects are likely not involved. That said, it is possible that ketones are preferentially utilized by skeletal muscle and that this results in the skeletal muscle-sparing effects that we observed. In fact, evidence to support this notion is the finding that skeletal muscle obtains 50%–85% of its fuel from ketones during fasting (37), thus high rates of ketolysis in skeletal muscle may preserve expression of ketolytic enzymes (as we observed), further supporting the energetic requirements of skeletal muscle anabolism. Based on this, we speculate that the muscle-sparing effects of ketone ester supplementation are not driven by increased caloric intake, but rather by the preferential utilization of ketones by skeletal muscle, helping to maintain anabolic processes and preserve muscle mass during semaglutide treatment.

Beyond our data supporting the idea that ketone therapy prevents semaglutide-induced deficits in skeletal muscle ketone metabolism, we also show that ketone therapy prevents semaglutide-induced reductions in the expression of genes encoding proteins essential for optimal mitochondrial function. Given that mitochondrial dysfunction is associated with, and possibly a driver of, increased atrophy in skeletal muscle (32, 33), it is not surprising that we also observed a significant increase in transcript levels of key atrophic regulatory proteins that govern skeletal muscle atrophy in skeletal muscle of semaglutide-treated mice. Additionally, since ketone therapy prevents these changes, we speculate that semaglutide-induced skeletal muscle mitochondrial dysfunction increases atrophy and that this contributes to the loss of skeletal muscle mass. That said, while our RNA-seq data support the idea that semaglutide impairs mitochondrial function and promotes skeletal muscle atrophy, we were unable to confirm these changes at the protein level for relevant proteins that regulate atrophy. However, given the large number of transcripts affected, individual protein changes may be modest, with cumulative minor effects driving the observed phenotype. Thus, although we do not present direct evidence of increased atrophy protein expression, our findings align with the physiological reduction in skeletal muscle mass observed in these mice. Conversely, it is also possible that changes in atrophy are not the main driver of semaglutide-induced loss of skeletal muscle, and that other, yet unidentified, pathways may be involved.

As anticipated, semaglutide treatment induced widespread changes in skeletal muscle transcript levels compared with controls, and notably, many of these changes were prevented by ketone ester co-therapy. Despite these numerous changes, we speculate that semaglutide-induced reductions in skeletal muscle BDH1 and SCOT expression, and the resulting decrease in mitochondrial ketone flux, may represent the initiating event driving muscle mass loss. We assume this based on a previous study that showed that the genetic ablation of BDH1 in skeletal muscle and reduced ketolysis were sufficient to prevent mitochondrial remodeling needed during times of caloric deficit, which was also associated with a reduction in skeletal muscle mass (14). Importantly, ketone therapy effectively prevented the semaglutide-induced decreases in skeletal muscle BDH1 and SCOT expression and thus maintained the machinery necessary to sustain ketone flux in skeletal muscle.

While it remains unknown whether the beneficial effects of ketone supplementation as an adjunct to semaglutide translate to humans, the findings reported herein have the potential to dramatically improve outcomes for patients taking GLP-1RA therapy, and therefore clinical trials to investigate this approach are warranted. Notably, a recent small trial has shown that a ketogenic diet combined with the dual GLP-1R and glucose-dependent insulinotropic polypeptide (GIP) receptor agonist tirzepatide assists with preserving lean mass and muscle strength in patients with obesity (38), suggesting that ketone ester therapy may also be effective in patients receiving semaglutide. That said, increased ketosis has been found to be associated with progressive insulin resistance in humans (23), and we do not know whether this untoward effect occurred in our study or could develop after longer durations of ketone ester treatment. Thus, future studies should investigate this possible negative consequence of ketone therapy in semaglutide-induced weight loss.

In addition to the issues raised above, before definitive conclusions are drawn about the benefits of ketone therapy for preserving muscle mass during semaglutide treatment, several additional factors warrant careful consideration. For instance, while we attribute our ketone ester’s ability to prevent skeletal muscle loss to an elevation in circulating β-hydroxybutyrate levels, it is also possible that other metabolic by-products of this ketone ester are involved. Indeed, in the gut, each molecule of bis-octanoyl (*R*)-1,3-butanediol is hydrolyzed to release 2 octanoic acid molecules, which undergo classical ketogenesis to produce β-hydroxybutyrate, and a single 1,3-butanediol, which is also converted to β-hydroxybutyrate via alcohol and aldehyde dehydrogenases. Thus, it is possible that 1,3-butanediol could be the active molecule responsible for preserving muscle mass. In addition, although numerous studies use a 60% fat diet to investigate semaglutide-induced weight loss in mice (6, 9, 39–42), there is the possibility that these effects would not have been observed if we had used a diet more typical of a Western diet (i.e., with a lower percentage of fat). Although this is a potential limitation of our study, we believe that this is unlikely, as we have previously shown that mice fed a high-fat, high-sucrose diet consisting of 45% fat show similar weight loss effects using semaglutide (22). Moreover, our studies were performed in male mice, so we cannot be certain that the beneficial effects of ketone therapy also occur in female mice, though these experiments are currently under way.

An interesting discrepancy between our study and the literature that warrants discussion is that we report that semaglutide decreases molecular markers in a pattern consistent with impairments in mitochondrial number and/or function. However, we did not directly measure mitochondrial function and thus cannot definitively conclude that mitochondria are dysfunctional. Rather, it is quite possible that semaglutide treatment results in less skeletal muscle mass independently of the mitochondria and this in turn results in less mitochondria. Alternatively, it is also possible that despite the observation that there are fewer mitochondria in skeletal muscle of semaglutide-treated mice, these mitochondria may have enhanced function, resulting in no observable decreases in ATP production. In fact, a recent study showed that 3 weeks of semaglutide treatment of obese mice resulted in improved mitochondrial OXPHOS efficiency in gastrocnemius muscle with no changes in OXPHOS subunit expression between groups (43). In a striking contrast, we observe a significant reduction in OXPHOS subunit expression in gastrocnemius muscle of semaglutide-treated mice compared with controls as well as reduced VDAC expression, indicating reduced mitochondrial number in skeletal muscle of semaglutide-treated mice. While the differences between that study and our own that may explain these divergent results are not yet completely understood, there are some differences between our models that may contribute to the discrepancies. For instance, in our study, mice were switched from a high-fat diet to a regular chow diet during the semaglutide treatment, but in the other study, they were maintained on a high-fat diet (43). As such, it is possible that the mice in our study experienced a greater caloric deficit than those in the previous study, and that this contributed to reduced mitochondrial number and possibly function. Additionally, the mitochondrial adaptations induced by the different diets during semaglutide treatment could also result in differences in mitochondrial biogenesis, coupling efficiency, substrate preference, and/or ROS production, any of which could account for the differences reported between these two studies. Similarly, our study involved treating mice with 30 nM/kg/d of semaglutide to induce weight loss, while the same degree of weight loss was observed in the other study using 3 nM/kg/d of semaglutide (43). While this discrepancy could lead one to conclude that the higher dose of semaglutide used in our study was more damaging to the skeletal muscle, the reported percentage lean muscle loss was comparable between studies, showing similar physiological responses, thus making this explanation less likely. Overall, we cannot provide evidence explaining the discrepancy between our study and the existing literature, and future investigations are needed to address this fully.

Overall, despite some limitations to our study, our data provide important insights into the magnitude, functional consequences, and potential mechanisms of semaglutide-induced skeletal muscle loss. We also present preclinical evidence demonstrating that ketone therapy can prevent these adverse effects, likely through multiple complementary mechanisms. Given the potential harmful consequences of semaglutide-induced muscle loss, our findings support investigating the muscle-preserving effects of ketone therapy in clinical trials involving semaglutide for weight loss. Because ketone therapy has already been evaluated in multiple clinical trials for other indications, in which it was shown to be safe and well tolerated (21), the rapid translation of this approach could meaningfully improve the health and well-being of millions of people using semaglutide and related GLP-1RAs.

## Methods

### Sex as a biological variable.

Because weight gain induced by a high-fat diet containing 60 kcal% fat is less in female mice than in male mice, only male mice were used in our study. Since our study exclusively examined male mice, it is unknown whether the findings are relevant to female mice.

### Experimental animals.

All experimental procedures involving mice were approved by the University of Alberta Institutional Animal Care and Use Committee and were conducted in accordance with the *Guide for the Care and Use of Laboratory Animals* (8th edition, National Academies Press, 2011) published by the US National Institutes of Health. The University of Alberta follows the principles outlined by the Council for International Organizations of Medical Sciences for biomedical research involving animals and complies with the guidelines established by the Canadian Council on Animal Care. Seven-week-old C57BL/6 male mice (sourced from Charles River Laboratories or The Jackson Laboratory) were housed under standard conditions (12-hour light/12-hour dark cycle) with ad libitum access to a standard chow diet and water. In most instances, results from C57BL/6N and C57BL/6J mice were identical in terms of the effects on body weight, fat mass, and lean mass in the semaglutide and the semaglutide plus ketone therapy groups. For this reason, many of the physiological parameters combined data obtained from both strains of mice. After a 1-week acclimatization period, mice were transitioned to a high-fat diet containing 60 kcal% fat (D12492I, Research Diets) for 15–17 weeks to induce obesity.

### Intraperitoneal glucose tolerance tests.

Mice were fasted for 5–6 hours beginning at 9 am, after which they received intraperitoneal injections of Dex 50 (RAFTER 8) (2 g/kg body weight). Blood glucose concentrations were determined from tail-tip blood samples using an ACCU-CHEK Advantage glucometer (Roche Diagnostics, Laval, Canada), as previously described (44). Measurements were obtained at baseline (0 minutes) and at 15, 30, 60, 90, and 120 minutes after glucose administration.

### Blood insulin and β-hydroxybutyrate measurements.

For blood insulin measurement, plasma samples were collected from mice after a 6-hour fast via tail-tip bleeding. Insulin levels were quantified using the STELLUX Chemi Rodent Insulin ELISA kit (ALPCO) according to the manufacturer’s instructions. Blood ketone levels were measured using the FreeStyle Precision Neo system (Abbott).

### Histological assessment.

Cross sections of the gastrocnemius muscle were collected immediately upon dissection and fixed in 10% formalin (catalog HT501128, Sigma-Aldrich) for 24 hours. Samples were subsequently processed at the Alberta Diabetes Institute Core Facility, University of Alberta. Briefly, tissues were embedded in paraffin and sectioned at a thickness of 5 μm. Sections were stained with Wheat Germ Agglutinin (catalog W11261, Fisher Scientific) following the manufacturer’s instructions. More than 5 randomly selected fields per section were imaged using an EVOS FL Digital Inverted Fluorescence Microscope (Fisher Scientific). Cross-sectional areas were quantified using ImageJ software (National Institutes of Health).

### Semaglutide and ketone ester administration.

To determine the optimal concentration of the ketone supplement in the drinking water that is capable of elevating circulating ketone levels in mice to physiologically relevant and safe levels comparable to those observed during fasting, preliminary dose-finding experiments were performed using Qitone [bis-octanoyl (*R*)-1,3-butanediol]. A range of concentrations were tested, and circulating β-hydroxybutyrate levels were quantified. Based on these trials, a concentration of 0.114 g/mL was identified as sufficient to achieve the desired elevation in β-hydroxybutyrate that remained in the physiological range of ketone concentrations. The ketone ester was provided to mice ad libitum and replaced twice daily to ensure stability and consistency of dosing. After induction of obesity through high-fat diet feeding, mice were transitioned to a standard chow diet to model the lifestyle modification implemented in the STEP1 clinical trial (1). Animals were subsequently randomized to receive 1 of 3 treatments for 3 weeks: vehicle control (PBS), semaglutide (30 nM/kg/d, intraperitoneally), or the combination of semaglutide and ketone ester (0.114 g/mL, provided in their drinking water) (Figure 1D).

### Body composition analysis.

Body composition was assessed using an EchoMRI body composition analyzer at baseline (week 0) and at 1, 2, and 3 weeks after treatment initiation.

### Measurements of caloric intake.

To measure food and fluid consumption and calculate total daily caloric intake, mice were single-housed in the Comprehensive Lab Animal Monitoring System (CLAMS; Columbus Instruments) for a total of 48 hours. Mice were placed in these cages at 7, 14, and 21 days after initiation of treatment. Food and fluid intake recorded during the second full light/dark cycle was used for analysis. While in the system, mice continued to receive their daily semaglutide injections, and the ketone ester drink was replaced twice daily. The CLAMS system recorded the change in weight of the food and drink. Multiplying the change in weight by the caloric content of the food (3.02 kcal/g) and drink (1.09 kcal/mL; density of the drink was determined to convert g to mL), total caloric intake of the mice during the 24-hour measuring period was determined.

### Rearing activity test.

To assess rearing behavior, each mouse was individually placed into a clean, empty cage without bedding. After a 1-minute acclimation period, the total number of rears was recorded over a 1-minute observation interval. A rear was defined as the mouse standing upright on its hind limbs with both forelimbs lifted off the ground.

### Endurance treadmill running test.

Exercise performance was evaluated using a rodent treadmill (Columbus Instruments). Mice were acclimated to the treadmill over 2 consecutive days with a 10-minute training protocol: 5 minutes at 8 m/min followed by 2 minutes at 9 m/min and 3 minutes at 10 m/min, with constant encouragement via tail stimulation with a soft-bristle bottle brush. For the exercise performance test, mice ran to exhaustion using the following protocol: 10 minutes at 10 m/min followed by incremental increases of 2 m/min every 5 minutes until exhaustion. Exhaustion was defined as the inability to continue running for 10 consecutive seconds despite encouragement. Total time to exhaustion (in seconds) was recorded. The test was conducted at baseline (week 0) and at 1, 2, and 3 weeks after treatment.

### Strength test.

Forelimb and total grip strength was assessed using a grip strength meter (Columbus Instruments). Mice were allowed to grasp a metal grid using their forelimbs and hind limbs. They were then briefly pulled away from the grid in the horizontal plane, and the absolute force exerted was recorded in grams. Trials were excluded if the mouse failed to grip with all limbs or made no effort to grip the grid. For each mouse, 5 consecutive measurements were recorded, and the average value was used for analysis.

### Immunoblot analysis.

Frozen tissue samples were homogenized following previously published methods (45), and protein concentrations were determined using the bicinchoninic acid (BCA) protein assay kit (catalog 23255, Pierce, Thermo Fisher Scientific). Approximately 15 μg of protein per sample was resolved by SDS-PAGE and transferred to a 0.2 μm nitrocellulose membrane (catalog 1704272, Bio-Rad). The following primary antibodies were used: anti–β-hydroxybutyrate dehydrogenase 1 (anti-BDH1; (catalog NBP1-88673, Novus Biologicals Toronto), anti–succinyl-CoA:3-ketoacid-CoA transferase (anti-SCOT1; catalog 12175-1-AP, Proteintech), anti-OxPhos Rodent WB Antibody Cocktail (catalog 45-809-9, Invitrogen), and anti–voltage-dependent anion channel (anti-VDAC; catalog 4661, Cell Signaling Technology). Protein bands were visualized using a ChemiDoc MP imaging system (Bio-Rad) with Clarity Max Western ECL substrate (catalog 1705062, Bio-Rad). Densitometric analysis was performed using Image Lab software (version 5.2.1, Bio-Rad). Total protein staining was carried out using the Pierce Reversible Protein Stain Kit for Nitrocellulose Membranes (catalog 24580, Thermo Fisher Scientific), following the manufacturer’s protocol.

### RT-qPCR.

Total RNA was extracted from frozen tissue samples using TRIzol reagent (Invitrogen), following the manufacturer’s instructions. Complementary DNA was synthesized using 5× All-In-One RT MasterMix (catalog G486, Applied Biological Materials), also according to the manufacturer’s protocol. RT-qPCR was performed using PowerUp SYBR Green Master Mix (catalog A25742, Applied Biosystems) in white 384-well plates on a LightCycler 480 system (Roche Life Science), as previously described (45). Primer sequences are listed in Supplemental Table 1 (supplemental material available online with this article; https://doi.org/10.1172/jci.insight.201810DS1) and were obtained from Integrated DNA Technologies. Gene expression was analyzed using the comparative Ct (2^–ΔΔCt^) method, with ribosomal protein L32 (*Rpl32*) or tyrosine 3-monooxygenase/tryptophan 5-monooxygenase activation protein ζ (*Ywhaz*) serving as the housekeeping gene.

### RNA sequencing.

Total RNA was extracted from frozen gastrocnemius samples using TRIzol reagent (Invitrogen), following the manufacturer’s instructions. Transcriptomic analysis was performed through the use of RNA sequencing (RNA-seq) in the School of Biomedical Engineering, Sequencing Core, University of British Columbia (Vancouver, British Columbia, Canada). Briefly, sample quality control was performed using the Agilent 2100 Bioanalyzer or the Agilent 4200 TapeStation. Qualifying samples were then prepared following the standard protocol for the Illumina Stranded mRNA Prep. Sequencing was performed on the Illumina NextSeq2000 with paired-end 59 bp × 59 bp reads. Sequencing data were demultiplexed using Illumina’s BCL Convert. Demultiplexed read sequences were then aligned to the *Mus musculus* (mm10) reference sequence using DRAGEN RNA application on Basespace Sequence Hub (https://supportdocs.illumina.com/SW/DRAGEN_v41/Content/SW/DRAGEN/TPipelineIntro_fDG.htm). Raw FASTQ data files, normalized counts, and metadata information can be found in the Gene Expression Omnibus (accession GSE326952).

### RNA-seq analysis.

RNA-seq data were processed via 1-way ANOVA (*P* < 0.05, fold change ≥ 1.5) (Supporting Data Values file) to evaluate transcript abundance across all 3 groups. For visualization, a triplot was constructed based on 3 pairwise log_2_-transformed expression ratios as previously described (46). These ratios were projected into 2-dimensional space using trigonometric transformations. Each gene was represented as a single point on the graph, with its *x* and *y* coordinates reflecting its relative abundance across all 3 conditions. The position of each point reflected the gene’s relative expression across all 3 conditions, enabling visualization of how transcripts were distributed among the treatment groups (the Supporting Data Values file contains all calculations). The filtered gene set was then clustered using the SRplot gene expression trend tool (47), which implements the Mfuzz algorithm (48), applying soft clustering to identify transcripts with similar expression trajectories. Five distinct clusters were created from the enriched dataset with membership scores. These clusters were used to color-code genes within the triplot (Supporting Data Values file). Gene Ontology (GO) analysis was performed on the lists of genes extracted from each cluster using Metascape (49). A minimum fold change ≥ 1.5 threshold was applied, and analyses included three GO categories: biological process, molecular function, and cellular component. Selective GO clustering (beta feature) was enabled to reduce redundancy and group similar terms. A minimum of 5 genes was used per GO term, and the background genome used for analyses was all genes detected in the RNA-seq data. Full GO analysis results for all clusters are provided in the Supporting Data Values file.

### Statistics.

Data are presented as the mean ± standard error of the mean (SEM). Comparisons between 2 groups were conducted using an unpaired, nonparametric Mann-Whitney test. For comparisons among 3 groups, 1-way ANOVA was performed followed by Tukey’s multiple-comparison test. *P* values less than 0.05 were considered statistically significant. Heatmaps were generated using row-wise *z* score normalization of expression values. Total normalized counts were compared using 1-way ANOVA followed by Tukey’s multiple-comparison post hoc test. *P* values less than 0.05 were considered statistically significant. All graphical and statistical analyses were carried out using GraphPad Prism version 10.

### Study approval.

The study protocols were approved by the University of Alberta Institutional Animal Care and Use Committee (AUP00003528), University of Alberta, Edmonton, Alberta, Canada.

### Data availability.

All data presented in this article are included in the Supporting Data Values file and are available upon request. RNA-seq data can be found in the NCBI’s Gene Expression Omnibus database (accession number GSE326952).

## Author contributions

YA, MAS, MN, RV, MAEG, EAK, MCPZ, DS, LK, RKS, ALT, DKR, DYML, YL, SOM, QS, and JLL conducted experiments. YA, MAS, and MN acquired data. MAS, RV, and SB analyzed data. YA, MAS, and JRBD designed research studies and wrote the manuscript. MF, RPF, and JRBD supervised trainees and edited the manuscript. YA, MAS, and MN devoted a comparable level of total effort to the project, with variations in their specific contributions to designing research studies, conducting experiments, acquiring data, analyzing data, and writing the manuscript. Thus, YA, MAS, and MN share the first authorship position. The order in which they are listed was assigned based on the recommendations made by the International Committee of Medical Journal Editors.

## Conflict of interest

The authors have declared that no conflict of interest exists.

## Funding support

- Grants from the Canadian Institutes of Health Research to JRBD.
- JRBD is a Canada Research Chair in Molecular Medicine.
- A Fellowship from the Women and Children’s Health Research Institute (WCHRI) of Alberta to YA.
- Canada Graduate Scholarship–Master’s Program to MAS.
- WCHRI Graduate Studentship to MAS.
- Alberta Diabetes Institute Graduate Studentship to MAS.
- Alberta Innovates Postdoctoral Fellowship to MN.

## Supplementary Material

Supplemental data

Unedited blot and gel images

Supporting data values

## Version 1 06/09/2026

In-Press Preview

## Version 2 08/10/2026

Electronic publication

## References

[1] Wilding JPH (2021). Once-weekly semaglutide in adults with overweight or
obesity. N Engl J Med.

[2] Jiao R (2025). Characterizing body composition modifying effects of a glucagon-like
peptide 1 receptor-based agonist: a meta-analysis. Diabetes Obes Metab.

[3] Arnold C (2024). After obesity drugs’ success, companies rush to preserve skeletal
muscle. Nat Biotechnol.

[4] Gonzalez Trotter D (2025). GDF8 and activin A are the key negative regulators of muscle mass in
postmenopausal females: a randomized phase I trial. Nat Commun.

[5] Heymsfield SB (2026). Bimagrumab plus semaglutide alone or in combination for the treatment of
obesity: a randomized phase 2 trial. Nat Med.

[6] Mastaitis JW (2025). GDF8 and activin A blockade protects against GLP-1-induced muscle loss
while enhancing fat loss in obese male mice and non-human primates. Nat Commun.

[7] Prado CM (2018). Implications of low muscle mass across the continuum of care: a narrative
review. Ann Med.

[8] Mozaffarian D (2025). Nutritional priorities to support GLP-1 therapy for obesity: a joint
Advisory from the American College of Lifestyle Medicine, the American Society for
Nutrition, the Obesity Medicine Association, and The Obesity Society. Am J Clin Nutr.

[9] Nunn E (2024). Antibody blockade of activin type II receptors preserves skeletal muscle
mass and enhances fat loss during GLP-1 receptor agonism. Mol Metab.

[10] Karasawa T (2025). Unexpected effects of semaglutide on skeletal muscle mass and
force-generating capacity in mice. Cell Metab.

[11] Schmidt MA (2025). Ketone therapy prevents semaglutide-induced loss of cardiac
mass. Eur J Prev Cardiol.

[12] Jeromson S Semaglutide impacts skeletal muscle to a similar extent as caloric
restriction in mice with diet-induced obesity. J Physiol.

[13] Stubbs BJ (2024). Daily consumption of ketone ester, bis-octanoyl (R)-1,3-butanediol, is safe
and tolerable in healthy older adults in a randomized, parallel arm, double-blind,
placebo-controlled, pilot study. J Nutr Health Aging.

[14] Williams AS (2024). Ketone flux through BDH1 supports metabolic remodeling of skeletal and
cardiac muscles in response to intermittent time-restricted feeding. Cell Metab.

[15] Chen J (2022). Mechanism of reduced muscle atrophy via ketone body
(D)-3-hydroxybutyrate. Cell Biosci.

[16] Lin C (2024). β-Hydroxybutyrate inhibits FOXO3a by histone H3K9 β-hydroxybutyrylation to
ameliorate stroke-related sarcopenia. J Funct Foods.

[17] Wang Q (2024). Histone β-hydroxybutyrylation is critical in reversal of
sarcopenia. Aging Cell.

[18] Weckx R (2022). Efficacy and safety of ketone ester infusion to prevent muscle weakness in
a mouse model of sepsis-induced critical illness. Sci Rep.

[19] Park SB, Yang SJ (2024). Ketogenic diet preserves muscle mass and strength in a mouse model of type
2 diabetes. PLoS One.

[20] Zhou Y (2023). Exploring the therapeutic potential of ethyl 3-hydroxybutyrate in
alleviating skeletal muscle wasting in cancer cachexia. Biomolecules.

[21] Soni S (2025). Therapeutic potential of ketone bodies on exercise intolerance in heart
failure: looking beyond the heart. Cardiovasc Res.

[22] Martens MD (2024). Semaglutide reduces cardiomyocyte size and cardiac mass in lean and obese
mice. JACC Basic Transl Sci.

[23] Hall KD (2021). Effect of a plant-based, low-fat diet versus an animal-based, ketogenic
diet on ad libitum energy intake. Nat Med.

[24] Falkenhain K (2025). Ketones and insulin: a paradoxical interplay with implications for glucose
metabolism. J Endocr Soc.

[25] Monsalves-Alvarez M (2020). β-Hydroxybutyrate increases exercise capacity associated with changes in
mitochondrial function in skeletal muscle. Nutrients.

[26] Cui H (2024). Identifying Acss1, Mtfp1 and Oxct1 as key regulators and promising
biomarkers of sarcopenia in various models. Gene.

[27] Zhang P (2025). Identification of potential shared core biomarkers in type 2 diabetes and
sarcopenia. Sci Rep.

[28] Byrne NJ (2020). Chronically elevating circulating ketones can reduce cardiac inflammation
and blunt the development of heart failure. Circ Heart Fail.

[29] Affourtit C, Carré JE (2024). Mitochondrial involvement in sarcopenia. Acta Physiol (Oxf).

[30] Varughese JT (2021). The role of voltage-dependent anion channel in mitochondrial dysfunction
and human disease. Cells.

[31] García-Poyatos C (2024). Cox7a1 controls skeletal muscle physiology and heart regeneration through
complex IV dimerization. Dev Cell.

[32] Delfinis LJ (2025). Perspectives on the interpretation of mitochondrial responses during
skeletal muscle disuse-induced atrophy. J Physiol.

[33] Kamarulzaman NT, Makpol S (2025). The link between mitochondria and sarcopenia. J Physiol Biochem.

[34] Souza ALG (2025). Biomarkers of skeletal muscle atrophy based on atrogenes evaluation: a
systematic review and meta-analysis study. Int J Mol Sci.

[35] Dogra C (2007). TNF-related weak inducer of apoptosis (TWEAK) is a potent skeletal
muscle-wasting cytokine. FASEB J.

[36] Hindi SM (2014). Regulatory circuitry of TWEAK-Fn14 system and PGC-1α in skeletal muscle
atrophy program. FASEB J.

[37] Rebello CJ (2025). From starvation to time-restricted eating: a review of fasting
physiology. Int J Obes (Lond).

[38] Schiavo L (2025). Preliminary evidence suggests that a 12-week treatment with tirzepatide
plus low-energy ketogenic therapy is more effective than its combination with a
low-calorie diet in preserving fat-free mass, muscle strength, and resting metabolic
rate in patients with obesity. Nutrients.

[39] Liu D (2022). Comparison of beneficial metabolic effects of liraglutide and semaglutide
in male C57BL/6J mice. Can J Diabetes.

[40] Duan X (2024). Semaglutide alleviates gut microbiota dysbiosis induced by a high-fat
diet. Eur J Pharmacol.

[41] Ren Q (2022). An effective glucagon-like peptide-1 receptor agonists, semaglutide,
improves sarcopenic obesity in obese mice by modulating skeletal muscle
metabolism. Drug Des Devel Ther.

[42] Yang L (2024). Semaglutide reduces cardiomyocyte damage caused by high-fat through
HSDL2. Drug Des Devel Ther.

[43] Choi RH (2025). Semaglutide-induced weight loss improves mitochondrial energy efficiency in
skeletal muscle. Obesity (Silver Spring).

[44] Koonen DP (2010). Alterations in skeletal muscle fatty acid handling predisposes middle-aged
mice to diet-induced insulin resistance. Diabetes.

[45] Soni S (2022). Exogenous ketone ester administration attenuates systemic inflammation and
reduces organ damage in a lipopolysaccharide model of sepsis. Biochim Biophys Acta Mol Basis Dis.

[46] Hussain FS (2023). Residual serum fibrinogen as a universal biomarker for all serotypes of
Myasthenia gravis. Sci Rep.

[47] Tang D (2023). SRplot: a free online platform for data visualization and
graphing. PLoS One.

[48] Kumar L, Futschik ME (2007). Mfuzz: a software package for soft clustering of microarray
data. Bioinformation.

[49] Zhou Y (2019). Metascape provides a biologist-oriented resource for the analysis of
systems-level datasets. Nat Commun.

