# Supplementary material for: Semaglutide-induced loss of skeletal muscle mass is blunted by co-administration of ketone esters
Source: JCI Insight. 2026 Jun 9;11(15):e201810. doi: 10.1172/jci.insight.201810 (PMC13464422; doi:10.1172/jci.insight.201810)
Supplement: Supplementary file 1 [file jciinsight-11-201810-s028.pdf]

Supplemental Table 1. List of primer sequences

Gene	Primer Sequence (5'-3')	
Atp5e	Fw	CAGGCTGGACTCAGCTACATC
	Rv	GTTCGCTTTGAACTCGGTCTT
Bdh1	Fw	AGGCTGTGACTCTGGATTTGGG
	Rv	CTGGATGGTTCTCAGTCGGTCA
Cox20	Fw	GAGAACACTCCATGTGCTCGAG
	Rv	CCAGCATCCTAAAGTCACCAGG
Cox7a1	Fw	GCTCTGGTCCGGTCTTTTAGC
	Rv	GTACTGGGAGGTCATTGTCGG
Fbxo32	Fw	GTTTTCAGCAGGCCAAGAAG
	Rv	TTGCCAGAGAACACGCTATG
Oxct1	Fw	GCATAAGGGGTGTGTCTGCT
	Rv	GCAAAATCTCCCGTGATGGC
Rpl32	Fw	GCTGCCATCTGTTTTACGG
	Rv	TGACTGGTGCCTGATGAACT
Smdt1	Fw	AGGAGAGGTGGCGATGTCTACA
	Rv	CACTCGGAGAAGACCAAAGGAC
Tnfrsf12a	Fw	GTGTTGGGATTCGGCTTGGT
	Rv	GTCCATGCACTTGTCGAGGTC
Trim63	Fw	GTGTGAGGTGCCTACTTGCTC
	Rv	GCTCAGTCTTCTGTCCTTGGA
Uqcr11	Fw	CTGGACTGGGTGCCTTACAT
	Rv	TGTCTTCCACAGGACTACGC
Uqcrq	Fw	CGCCTTCCCAAGCTATTTCAGC
	Rv	CGACTGCTCAAACTCCTGGTTG
Ywhaz	Fw	TGAGCTGTCGAATGAGGAGAG
	Rv	CCTCCACGATGACCTACGG

Fw: Forward. Rv: Reverse
