# Supplementary material for: Semaglutide-induced loss of skeletal muscle mass is blunted by co-administration of ketone esters
Source: JCI Insight. 2026 Jun 9;11(15):e201810. doi: 10.1172/jci.insight.201810 (PMC13464422; doi:10.1172/jci.insight.201810)
Supplement: Supplementary file 2 [file jciinsight-11-201810-s029.pdf]

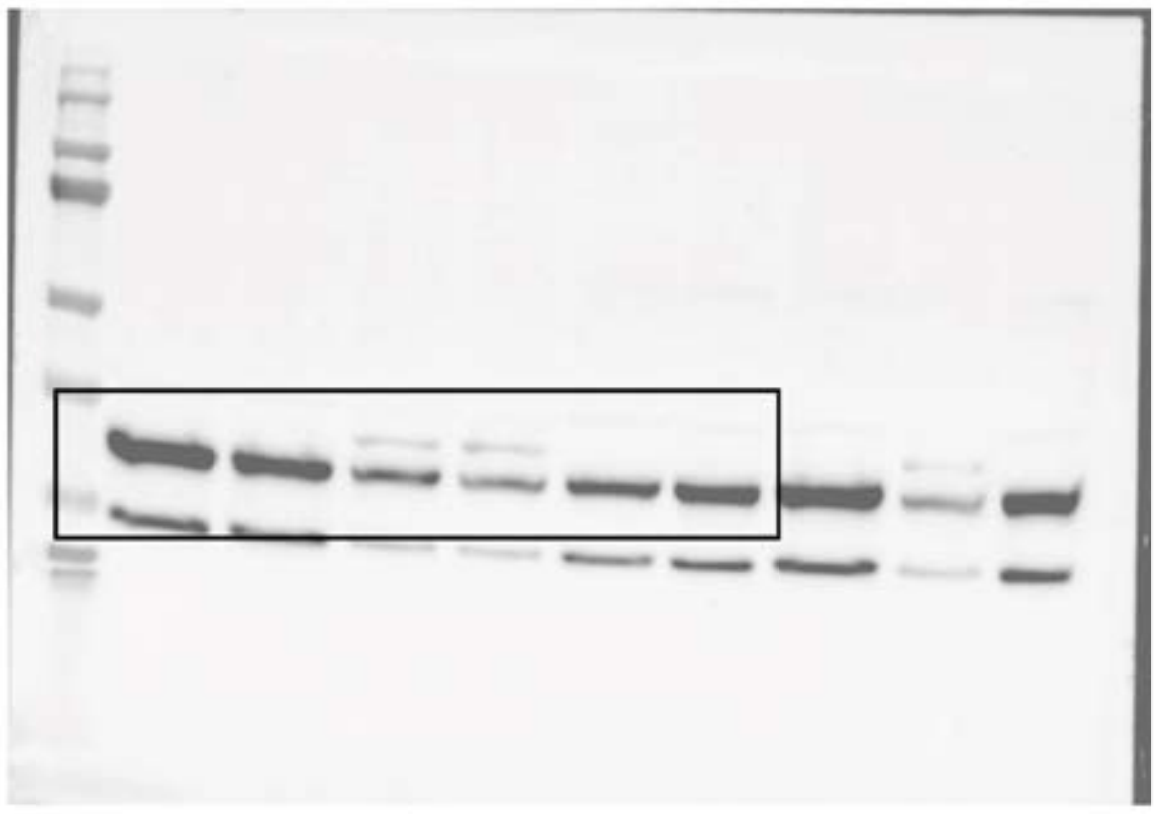

Full unedited blot for Figure 4C, BDH-1

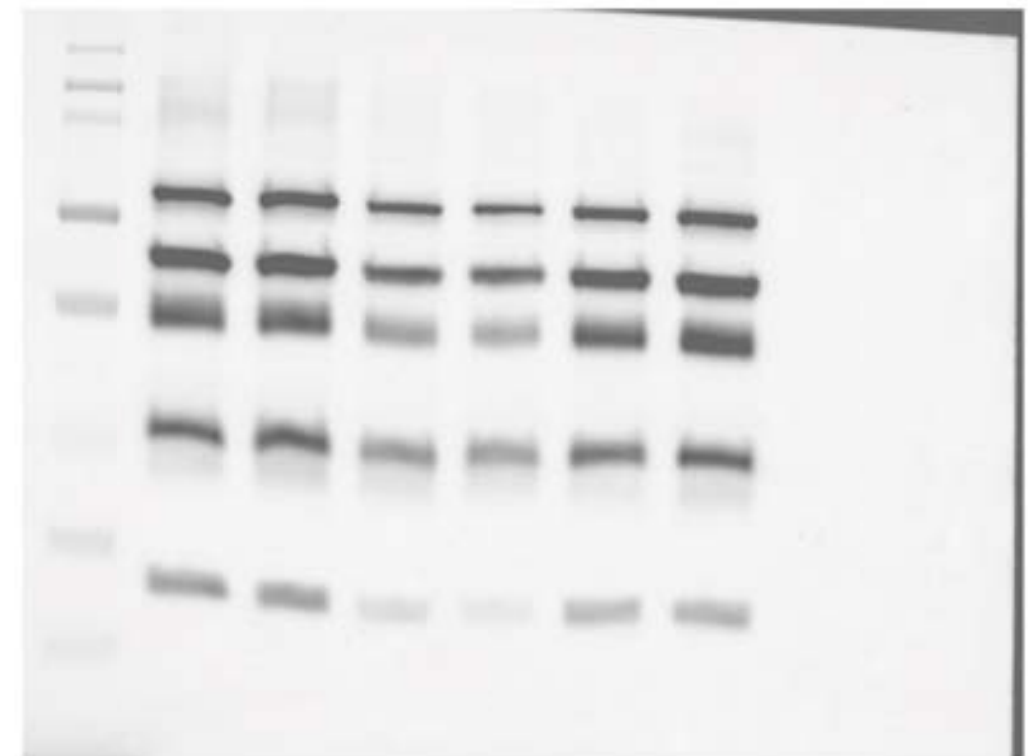

Full unedited blot for Figure 6A, OXSPHOS

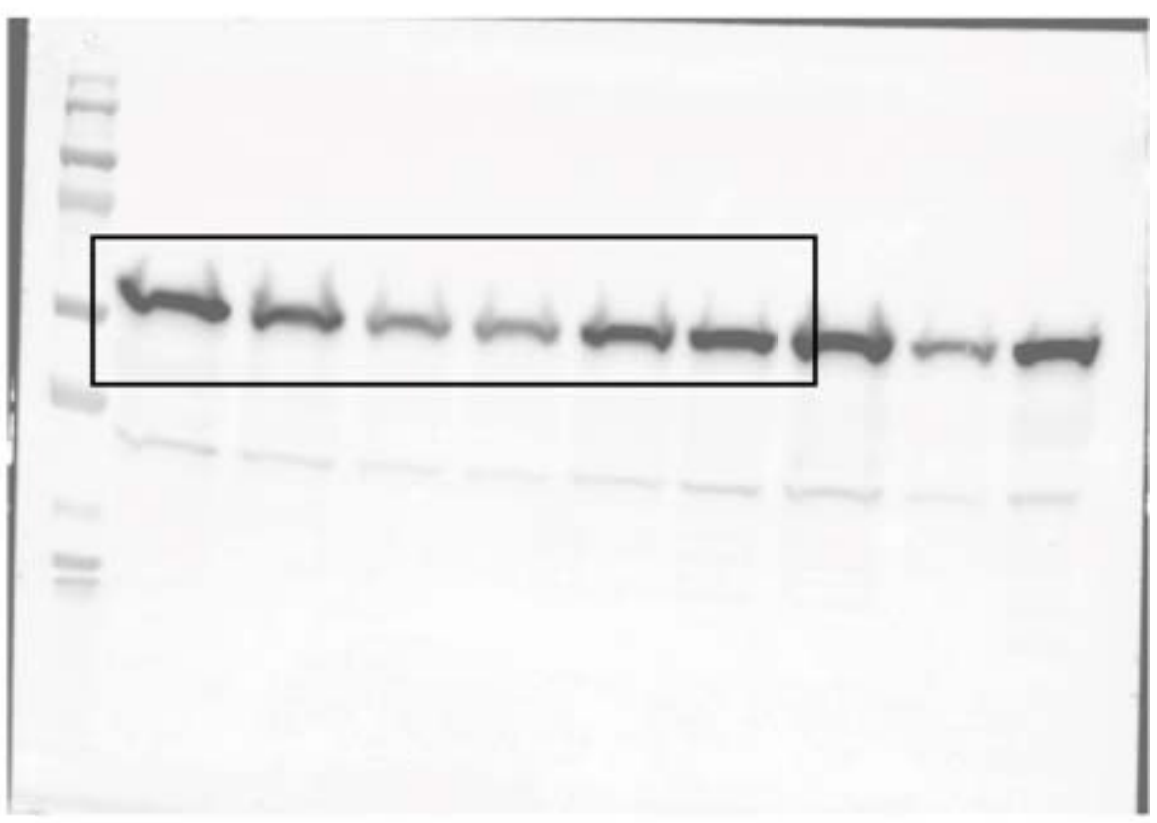

Full unedited blot for Figure 4C, SCOT

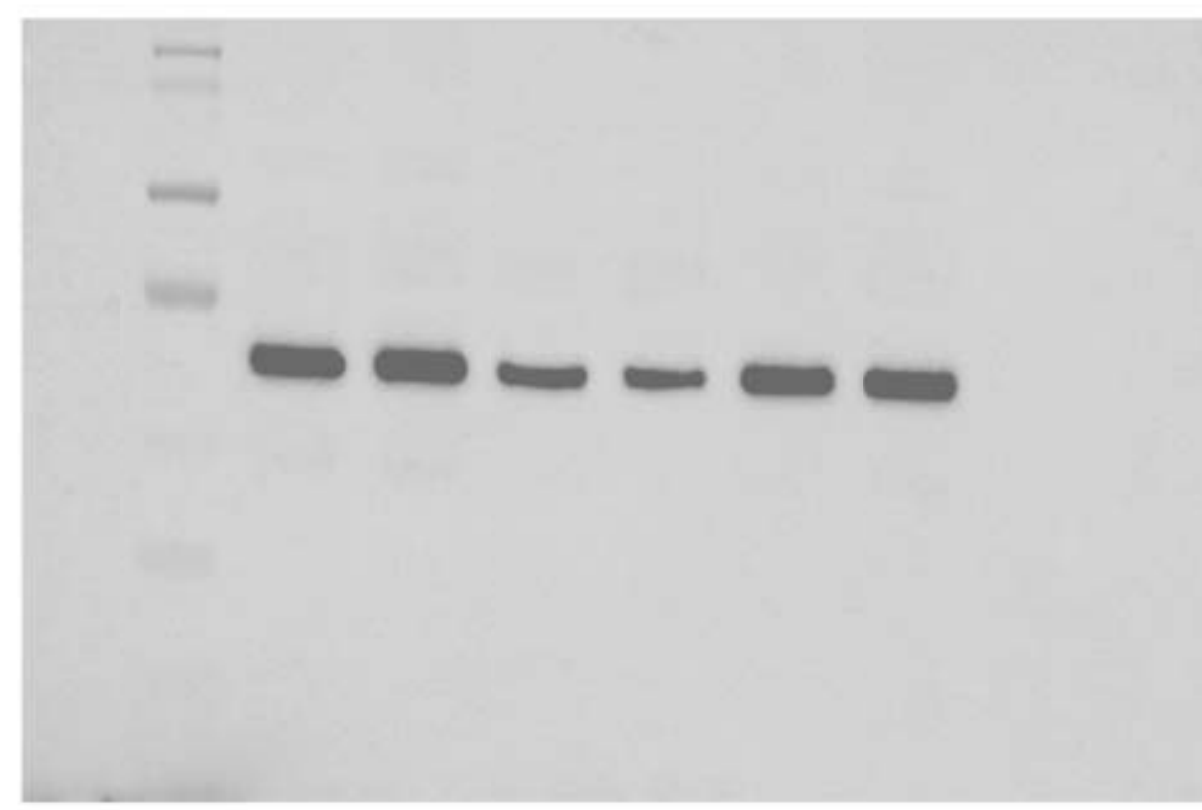

Full unedited blot for Figure 6A, VDAC

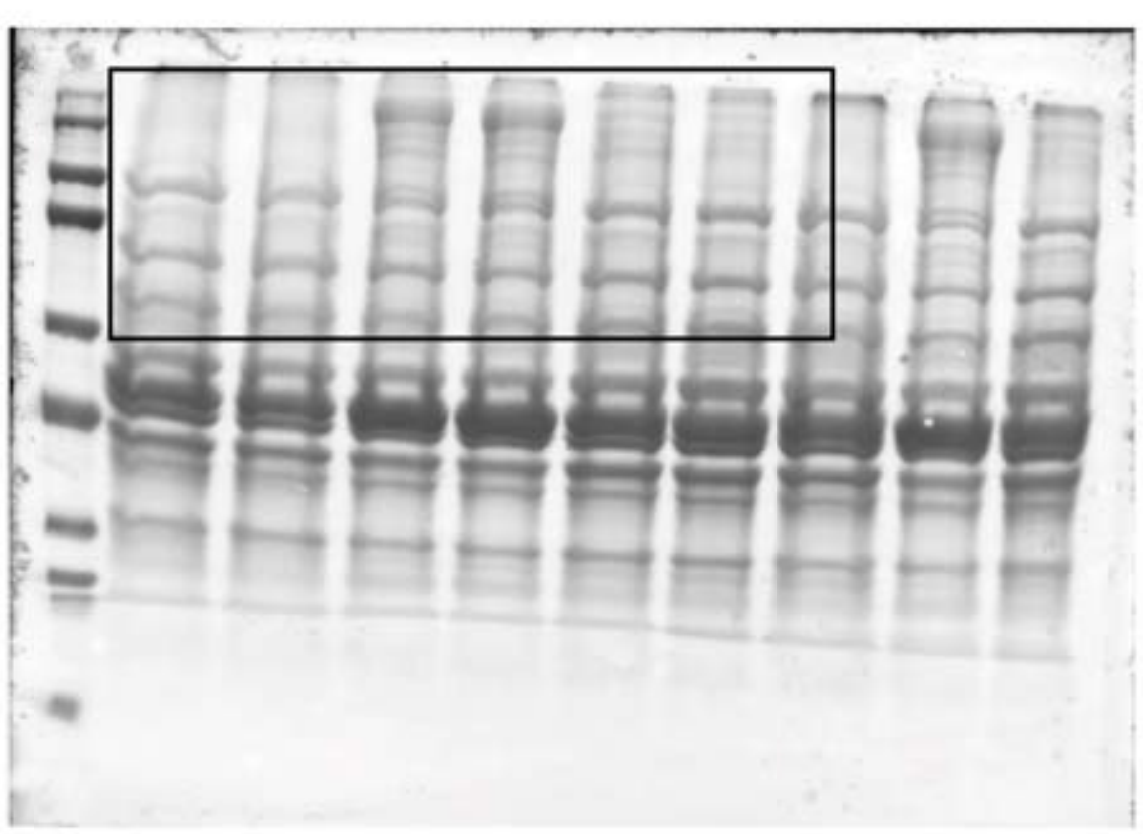

Full unedited blot for Figure 4C, Total protein staining

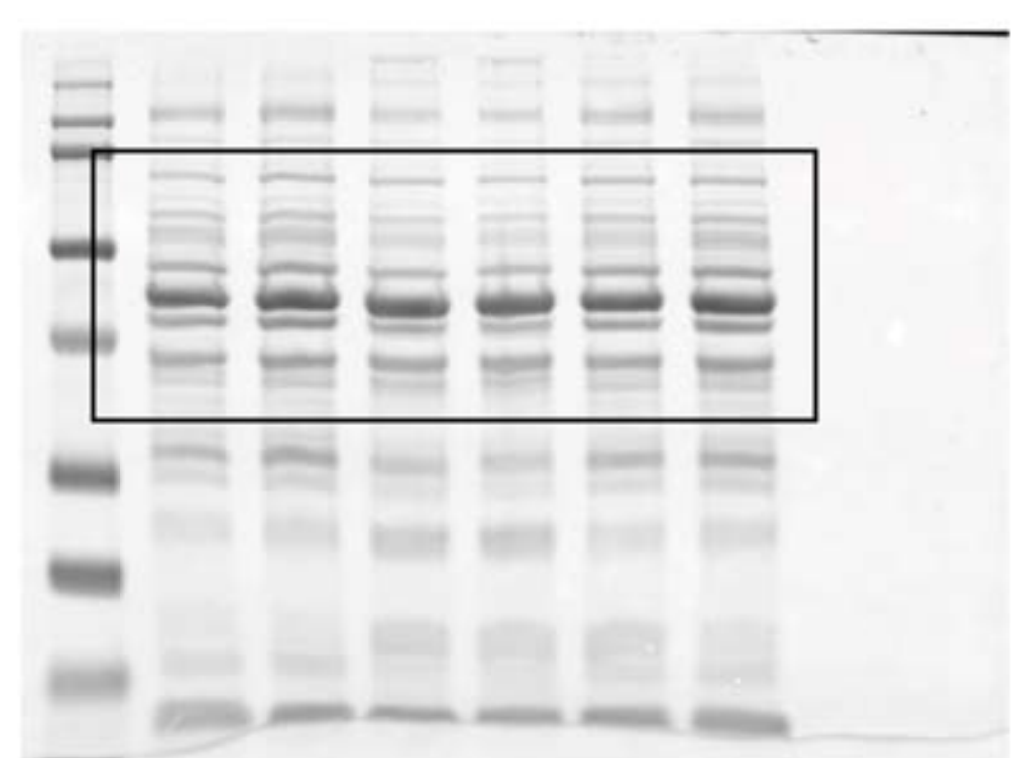

Full unedited blot for Figure 6A, Total protein staining
